# Quantum Chemical Calculation for Intermolecular Interactions of Alginate Dimer-Water Molecules

**DOI:** 10.3390/gels8110703

**Published:** 2022-10-31

**Authors:** Daru Seto Bagus Anugrah, Laura Virdy Darmalim, Muhammad Rifky Irwanto Polanen, Permono Adi Putro, Nurwarrohman Andre Sasongko, Parsaoran Siahaan, Zeno Rizqi Ramadhan

**Affiliations:** 1 Biotechnology Study Program, Faculty of Biotechnology, Atma Jaya Catholic University of Indonesia, BSD Campus, Tangerang 15345, Indonesia; 2 Food Technology Study Program, Faculty of Biotechnology, Atma Jaya Catholic University of Indonesia, BSD Campus, Tangerang 15345, Indonesia; 3 Department of Physics, Faculty of Science, Universitas Mandiri, Subang 41211, Indonesia; 4 Department of Chemistry, Faculty of Science and Mathematics, Diponegoro University, Semarang 50275, Indonesia; 5 Department of Chemistry, Pukyong National University, Busan 48513, Korea; 6 School of Chemistry, University of New South Wales, Sydney, NSW 2052, Australia

**Keywords:** alginate, density functional theory, NBO, QTAIM, water

## Abstract

The abundance of applications of alginates in aqueous surroundings created by their interactions with water is a fascinating area of research. In this paper, computational analysis was used to evaluate the conformation, hydrogen bond network, and stabilities for putative intermolecular interactions between alginate dimers and water molecules. Two structural forms of alginate (alginic acid, alg, and sodium alginate, SA) were evaluated for their interactions with water molecules. The density functional theory (DFT-D3) method at the B3LYP functional and the basis set 6-31++G** was chosen for calculating the data. Hydrogen bonds were formed in the Alg-(H_2_O)_n_ complexes, while the SA-(H_2_O)_n_ complexes showed an increase in Van der Walls interactions and hydrogen bonds. Moreover, in the SA-(H_2_O)_n_ complexes, metal-nonmetal bonds existed between the sodium atom in SA and the oxygen atom in water (Na…O). All computational data in this study demonstrated that alginate dimers and water molecules had moderate to high levels of interaction, giving more stability to their complex structure.

## 1. Introduction

Over the years, hydrogels have become an object of interest to be explored due to their massive functional properties, particularly for many fields of industries [1,2]. There are abundant definitions of hydrogel, which mostly describe hydrogels as a group of polymeric materials with a cross-linked network developed by one or more monomers’ modest reactions. Three-dimensional hydrogel networks are the main part providing hydrogels with the ability to hold large amounts of water and build hydrophilic structures [3]. The capability of having significant water content causes this material to carry a degree of flexibility identical to natural tissue. Its hydrophilic functional groups adhered to the polymeric backbone are responsible for absorbing water, while the cross-links between network chains take a role in assuring hydrogels’ resistance to dissolution. The mass fraction of water in hydrogels can be much greater than the mass fraction of the polymer in the hydrogel; this is also known as being in a swollen state, depending not only the polymer or polymers’ properties but also the network joints’ nature and density. Based on its type of cross-linking and whether it is a chemical or physical nature, permanent junctions result from chemically cross-linked networks, while transient junctions are promoted by physical networks through the entanglements of polymer chains or physical interactions, for instance, hydrogen bonds, hydrophobic or ionic interactions [4]. 

Both natural and synthetic materials are examined, mainly to be assigned as considerable candidates for hydrogels. The most commonly used natural hydrogels in the biomedical field, especially for regenerative medicine, are alginates due to their biocompatibility and low level of toxicity [5]. Moreover, these polysaccharides derived from brown algae extraction are also widely applied as gels and thickening agents in the manufacturing industries [6,7]. Alginate is used to manufacture hydrogels with a high ability to bind water, drugs, and protein, so it is widely used in drug and protein delivery systems [8,9,10,11,12] and wound management [13,14,15]. Their abundant existence in the wild allows them to have a low extraction cost. (1 → 4) glycosidic bridges link β-D-mannuronic acid (M) and α-L-guluronic acid (G), which build the linear binary copolymers [16]. Alginate is an unbranched copolymer composed of mannuronic acid (M block) and guluronic acid (G block) arranged in an irregular pattern with varying amounts of GG, MG, and MM blocks. M residues are linked at ^4^C_1_ by diequatorial linkages, while G residues are linked via diaxial links at ^1^C_4_ [6,17]. Globally, alginate is classified into two structural forms: alginic acid and sodium alginate. Alginic acid possesses a carboxylic acid functional group (−COOH), while sodium alginate is its sodium salt. The structure of alginic acid makes it difficult to dissolve, but the form of sodium alginate makes it highly soluble in water [17]. 

Currently, theoretical approaches based on computational chemistry can describe interactions that occur at the molecular or atomic level. Because of advancements in molecular modeling, computational chemistry applications are now widely employed to aid in many types of research in domains, such as health, energy, industry, environmental technologies, and pharmaceuticals. Rojas et al. investigated the effect of sodium and calcium cations on the structure of alginate [18]. Density functional theory (DFT), one of the theoretical methods of computing, has reignited interest in the nature and location of interactions between nanomaterials and biomolecules. The interaction of alginate (with a GG block structure) with bivalent metals, such as Cu^2+^, Mn^2+^, Ca^2+^, and Mg^2+^, has been studied theoretically using DFT [19]. However, there has been no research on computational studies explaining how the interactions between the alginate and water molecules occur at the molecular or atomic level.

In order to reveal the explanation with a theoretical approach using the computational chemistry of alginic acid-water, density functional theory with Grimme D3 dispersion correction (DFT-D3) was employed in this study. DFT was used due to the inclusion of the electron correlation’s effects with the determination of the molecular system’s properties and electron density. In this study, B3LYP-D3/6-31++G** was used to obtain more precise calculations in the actual experiment [20]. In the most stable molecular associations in Alg-(H_2_O)_n_ and SA-(H_2_O)_n_-water complexes, intermolecular interactions were analysed not only with the DFT method but also the natural bond orbital (NBO) and atoms-in-molecules (AIM) methods, which are mainly performed for representing the organic compounds’ hydrogen bonds in aqueous solutions [21]. NBO analysis can observe the intra- and intermolecular hydrogen bonds. Protons that are given to one accepting orbital for the determination of the hydrogen bond’s direction occur in intramolecular hydrogen bonds. Yet, protons that are moved in a comparable number for both lone pairs from acceptors happen in intermolecular hydrogen bonds [22]. Furthermore, the highest occupied molecular orbital (HOMO) and lowest unoccupied molecular orbital (LUMO) energies were also added to better understand these intermolecular interactions. Eventually, this study could disclose the interaction between alginate and water molecules or their atoms and support the idea of the use of alginate for hydrogel materials.

## 2. Results and Discussion

### 2.1. Optimized Structures of Alginate and Water Molecules

**Figure 1:**
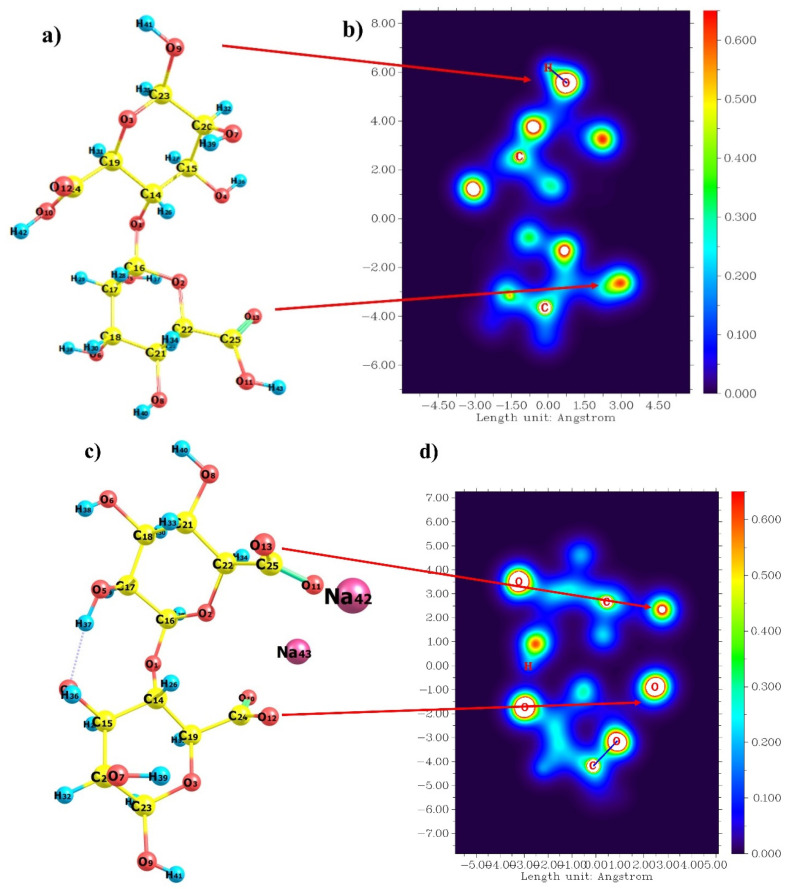
Optimized structure and molecular electrostatic potential of dimer alg (**a**,**b**), and dimer SA (**c**,**d**).

Optimizing a molecule structure was the first step before evaluating other parameters. The NWChem program was applied to obtain a stable molecule and minimum energy of dimer alginate and water structures [23]. The optimized structures of alginic acid (Alg) and sodium alginate (SA) are presented in Figure 1. Dimer Alg and SA structure were chosen to represent the polymer due to the fact that the interaction energy and enthalpies between dimers, trimers, and oligomers with water are almost similar [24,25,26,27]. Researchers also used dimer structures to evaluate the polymer [24,26,28,29]. G blocks (GG) of the alginate structure are stiffer compared to alternate blocks (GM) and M blocks (MM) due to their diaxial connection, and hence, they are more soluble at lower pH [16,30]. Thus, in this study, M blocks are used to examine the flexibility of alginate as it interacts with water molecules.

The carboxylic acid and hydroxyl groups in the alginic acid and the alginate structure are highly polar, making interactions with water molecules possible. This statement is supported by Figure 1 and Figure S1, which show the molecular electrostatic potential of alginic acid, alginate and water. The blue side to the red side shows the least to the greatest total density electron. The white area suggests that electron density exceeded the upper limit of the color scale (0.60), which meant that it was strongly negative. The MEP data demonstrate that the carboxylate group in the alginate form is more polar than the carboxylate group in the alginic acid form, as evidenced by the bigger white region. In the SA structure, metal-nonmetal interactions occur from Na atoms (Na42 and Na43) and oxygen atoms in the carboxyl group (O12 and O13). Furthermore, the polarity value was evaluated using total dipole moment, which revealed that SA had a greater value than Alg (6.83 debye and 4.03 debye, respectively) (Table S1). Larger dipole moments suggest higher positive and negative charges, implying that the molecule is more polar [31]. Alginate’s ionized carboxyl group is used to create polyelectrolyte complexes that are used in biological, pharmaceutical, and agricultural applications [32,33,34,35].

The conformation of alginate can be identified by its dihedral angle (χ) and torsion angles phi (φ) and psi (ψ) [32]. The considered alginate conformation presents a value for χ of 48.964° (Table 1). The value of χ could identify the conformation of both pyranose rings. The planarity of disaccharides, oligosaccharides, and alginate polysaccharides can be determined by φ and ψ. The values of φ and ψ were calculated between the bonds from each cyclic of the disaccharide moiety to its glycosidic oxygen atom [36]. The presence of water molecules greatly affected the conformation of the Alg and SA, as indicated by changes in χ, φ, and ψ when the number of water molecules increased (Table 1, Figure S2). These findings support the results of the study that alginate has conformational flexibility, which is an important factor in influencing the tendency of alginate to form macroscopic material such as a hydrogel [37,38]. Table 1 also shows that the molecular surface volume of Alg and SA increases with the increasing amount of water in the complex, which hints at the large swelling in water for hydrogel formation. The molecular volume of the SA-H_2_O complexes is larger than that of the Alg-H_2_O complexes due to the presence of metal atoms (sodium, Na). The structural deformation degree of Alg and SA following complexation was estimated using deformation energy $\left(E_{\mathrm{def}}\right)$ (Table S1). Because of the presence of water, the deformation energy of SA is higher than Alg (29.5 against 24.1 kcal/mol). These findings confirmed that water molecules interact with SA more strongly than the Ch structure. This is in accordance with the experimental results, which found that alginic acid is a water-insoluble polysaccharide, whereas the sodium form (SA) is a water-soluble polysaccharide [17,39]. In addition, previous research reports have shown that alginate, as a hydrogel substance, can interact strongly with water and has a high swelling ratio of more than ten times its dry mass [11,40].

The addition of water molecules also changed the structure of alginic acid and sodium alginate, as seen in Table 2 and Figure S3. Dissociation of alginic acid molecules happened as the water molecule number increased, which was presented with the longer bond lengths of O7-H39 and O11-H43, which were up to 1 Å. The same results were also reported by previous researchers [41,42]. On the other hand, Table 2 showed that sodium’s hydration process of carboxylic sodium salt occurred in addition to two water molecules, which was proved by the fact that the distance between O and Na was longer than 2.3 Å. These results evaluated the interactions that occur in alginate at low and high pH. Under acidic conditions, the alginate backbone’s carboxylate groups become protonated and form hydrogen bonds between the alginate chains. However, under basic conditions, alginate in carboxylate sodium salt will tend to form a sol phase [43,44,45].

### 2.2. NBO Analysis

Usually, the bonding interaction of hydrogen also consists of natural orbital to assure the strength of the interaction bond, which is investigated by NBO analysis. The result could also support the strength of the hydrogen bonding interaction that was predicted by the energy interaction [26,27]. NBO analysis is also used not only for investigating the intramolecular and intermolecular bonding interaction but also the charge transfer or conjugative interactions that happen in a molecular system. The stabilizing energy (E^(2)^) for donor-acceptor interaction represents the electron delocalization from the lone pair (LP) orbital, the occupied bond to unoccupied antibonding (BD*) orbital, the occupied bond to unoccupied bonding (BD), the number of cores (CR), and Rydberg (RY). The donor-acceptor interaction becomes more exhaustive when the stabilizing energy (E^(2)^) is greater [46]. Thus, the NBO analyses results of the formation of intermolecular bonds within alginic acid or sodium alginate and water molecules are shown in Tables S3 and S4. These results indicate that those complexes were all stabilized by hydrogen bonds, and the stabilization of their molecular arrangements were contributed by the hydrogen bond as well as the oxygen from both alginate and water molecules.

The O_5_ lone pair donor orbital that had an interaction with the O46-H44 anti-bonding orbital (LP(1)O_5_ → BD*(O46-H44) and LP(2)O5 → BD*(O46-H44)) appeared in all alginate-water complexes as well as the O_13_ lone pair that interacted with the O46-H44 anti-bonding orbital (LP(1)O_13_ → σ*(O46-H45) and LP(2)O13 → σ*(O46-H45)). The intermolecular charge transfer disclosed by the LP(1)O13 → BD*(O46-H45) and LP(1)O13 → BD*(O54-H55) interactions that occurred in the alginate-(H_2_O)_3_ complex was stabilized by the amount of energy, 2.98 kcal/mol and 3.67 kcal/mol, respectively. Meanwhile, the intermolecular charge transfer assigned by the same interaction as mentioned before also existed in the alginate-(H_2_O)_4_ complex with energies of 3.97 kcal/mol and 2.42 kcal/mol, respectively, used to stabilize the interaction, as well as in the alginate-(H_2_O)_5_ complex with energies of 2.14 kcal/mol and 7.22 kcal/mol, respectively. The energy of the second-order perturbation of LP(2)O46 → σ*(O47-H49) interaction that appeared in all alginate-(H_2_O) complexes except with one water molecule showed that their values were increasing along with more water molecules added to the complex, with the highest energy found in the Alg-(H_2_O)_5_ complex at 24.73 kcal/mol. On the other hand, LP(2)O54_54_ → BD*(O_11_-H_43_), which has an energy of about 46.21 kcal/mol, proved to be the strongest and highest hydrogen bond interactions among all complexes of the alginate and water molecules.

The protons could also be transferred easier with stronger hydrogen bonds [27], which implies that the more molecules of water exist in the alginate-water complex, the greater the energy that could contribute to the stability of the hydrogen-bonded structure. Thus, these NBO analyses of alginate-water complexes provided the idea of alginate for the application as a hydrogel that has the ability to absorb water that comes up from hydrophilic functional groups linked to the polymeric backbone and could swell due to the water [4]. Furthermore, a significant fraction of water with its structure could be restrained by the hydrogel. These hydrogel characteristics correspond to the results gained from the NBO analysis, which also demonstrates the structure of the water is not broken by alginate, despite water-water interactions affording the stabilization of the alginate-water mixture.

According to the NBO results, in particular, the LP(2)O46 → BD*(O_47_-H_49_) interaction formed in the Alg-(H_2_O)_2,_ Alg-(H_2_O)_3,_ Alg-(H_2_O)_4,_ and Alg-(H_2_O)_5_ complexes resulted in shorter interaction distance between O46 to O47-H49 due to the higher stabilizing energies (Figure S3 and Table S3, respectively). The same event was also found between the lone pair O47 and the antibonding orbital of O7-H39, which showed that the shorter its distance, the more stabilizing energy that occurred. Nevertheless, increasing water molecules could enhance the hydrogen bond between the water and alginate. Moreover, the more water molecules existed, the more strongly water interacted with the hydrophile group of other alginates, e.g., O46 with H49. As a result, these provided new insight into identifying which group is more likely to be hydrophilic and also demonstrated that the majority of alginate’s hydrophile groups could have interactions with water molecules as well as raise the water uptake. In the NBO analysis, the stabilization energy of sodium alginate was also evaluated (Table S4). According to the NBO analysis, some of the stabilization energy of Na bonds in the sodium alginate…water interaction is more than 50 kcal/mol, which was bigger than the alginate…water interaction that implied the sodium alginate…water has stronger interaction. There was the strongest charge transfer in the SA-(H_2_O)_5_ complex, which displayed the highest value of stabilization energy for the interaction at LP(1)O12 → RY(12) Na58 (104.81 kcal/mol). Rodríguez also evaluated interactions of alginate with bivalent metals and revealed the highest stabilization energy was 50.57 kcal/mol for a metal-nonmetal interaction (Mn..O) [19]. Their results indicated Alg-(H_2_O)_n_ complexes were stabilized by hydrogen bonds, while SA-(H_2_O)_n_ complexes were stabilized by metal-nonmetal interactions and hydrogen bonds.

### 2.3. Quantum Theory Atom in Molecule (QTAIM)

A saddle point of electron density located between two atoms, the donor group’s hydrogen atom and the acceptor atom that created a chemical bond, is called a bond critical point (BCP) [21]. Figure S4 illustrated BCPs on the interaction of the Alg-(H_2_O)_n_ complexes and SA-(H_2_O)_n_ complexes. The result of all parameters that are shown in Tables S5 and S6 were obtained by the multiwfn software.

Every complex showed a BCP that could be considered a weak covalent interaction or strong electrostatic bond due to the fact that both of their $\nabla^{2}\rho$ and H_(BCP)_ values were positive (Tables S5 and S6). In Alg-H_2_O, the positive values of $\nabla^{2}\rho$ and H_(BCP)_ were obtained from BCP with an index of 69. In alginate-(H_2_O)_2,_ the positive values of $\nabla^{2}\rho$ and H_(BCP)_ were obtained from BCP with indexes of 61, 108, 83, and 98. In alginate-(H_2_O)_3,_ the positive value of $\nabla^{2}\rho$ and H_(BCP)_ were obtained from BCP with indexes of 109, 118, 89, 77, 100, and 84. In alginate-(H_2_O)_4,_ the positive value of $\nabla^{2}\rho$ and H_(BCP)_ were obtained from BCP with indexes 77, 88, 124, and 118. In the alginate-(H_2_O)_5_ complex, the positive values of $\nabla^{2}\rho$ and H_(BCP)_ were obtained from BCP with indexes of 114, 80, 127, 125, and 75. Other BCPs in all complexes could be referred to as medium-strength or partially covalent bonds as a result of the positive values of $\nabla^{2}\rho$ and negative values of H_(BCP)_. Their |V/G| parameters also exhibited values between 1 and 2, which meant the interaction could be classified as medium interactions or medium hydrogen bonds as well, except for some of them that were mentioned before as having weak covalent interactions due to the positive values of $\nabla^{2}\rho$ and H_(BCP)_ and an alginate-(H_2_O)_4_ BCP index of 113. The complex of alginate-(H_2_O) with an index of 69_,_ alginate-(H_2_O)_2_ with indexes of 61, 108, 83, and 98, alginate-(H_2_O)_3_ with indexes of 109, 118, 89, 77, 100, and 84, alginate-(H_2_O)_4_ with indexes of 77, 88, and 118 and alginate-(H_2_O)_5_ with indexes 114, 90, and 127 had ratios of |V/G| < 1, which indicated a weak interaction between the alginate and water molecules existed there. The hydrogen bond energy (E_HB_) values belong to the indexes 69 (alginate-(H_2_O)), 61, 108, 83, 98 (alginate-(H_2_O)_2_), 109, 118, 89, 77, 100 (alginate-(H_2_O)_3_), 77, 88, 118 (alginate-(H_2_O)_4_), 114, 80, and 127 (alginate-(H_2_O)_5_) represented under 4 kcal/mol that refer to weak hydrogen bonds. Nevertheless, the rest of the bond critical points had an E_HB_ value of about 4–20 kcal/mol, which demonstrated that the interaction between alginic acid and water molecules had a moderate hydrogen bond. The BCPs for the interaction between SA and water are shown in Table S6. Overall, the data showed that the interaction was categorized as strong interactions. Values of $\nabla^{2}\rho$ and H_(BCP)_ revealed electrostatic interactions in the SA-(H_2_O)_n_ complexes. These QTAIM results supported the idea of alginate as a hydrogel material due mostly to the fact that its interaction with water was built by moderate to strong intermolecular bonds.

### 2.4. HOMO-LUMO Energy Analysis

HOMO-LUMO energy was calculated to obtain the reactivity of electron transfer. The capacity of a molecule to donate an electron is represented by E_HOMO,_ and the ability to accept an electron is staged by E_LUMO_. Thus, an increased HOMO energy value shows that more molecules tend to donate an electron to the acceptor. On the other hand, a decreasing LUMO energy value signifies that the molecule tends to take up more electrons. The energy difference between HOMO energy and LUMO energy is well known as the HOMO-LUMO energy gap $\left(\Delta E\right).$ The ΔE can be deployed to predict the chemical species’ strength and stability [26,47,48]. A small ΔE corresponds to a molecule that is likely to transfer electrons and be more reactive. In other words, it tends to be more unstable. The electronegativity information also relates to the electrophilicity of the molecule. Thus, the molecule can be said to be more electrophilic when it has a higher electronegativity [27]. The results of the HOMO-LUMO energy of Alg-(H_2_O)_n_ and SA-(H_2_O)_n_ complexes are sdisplayed in Table 3.

According to the values in Table 3, the DFT approach gives the ΔE value for Alg and SA as 6.84 and 6.43, respectively. The ΔE value of SA was smaller than Alg. The result indicated that the alginic acid structure had a high stabilization and rigid form compared with the sodium alginate structure. The presence of water molecules could decrease the energy gap of HOMO-LUMO in Alg and SA complex structures.

### 2.5. Reduced Density Gradient (RDG) and Non-Covalent Interaction (NCI) Analysis

**Figure 2:**
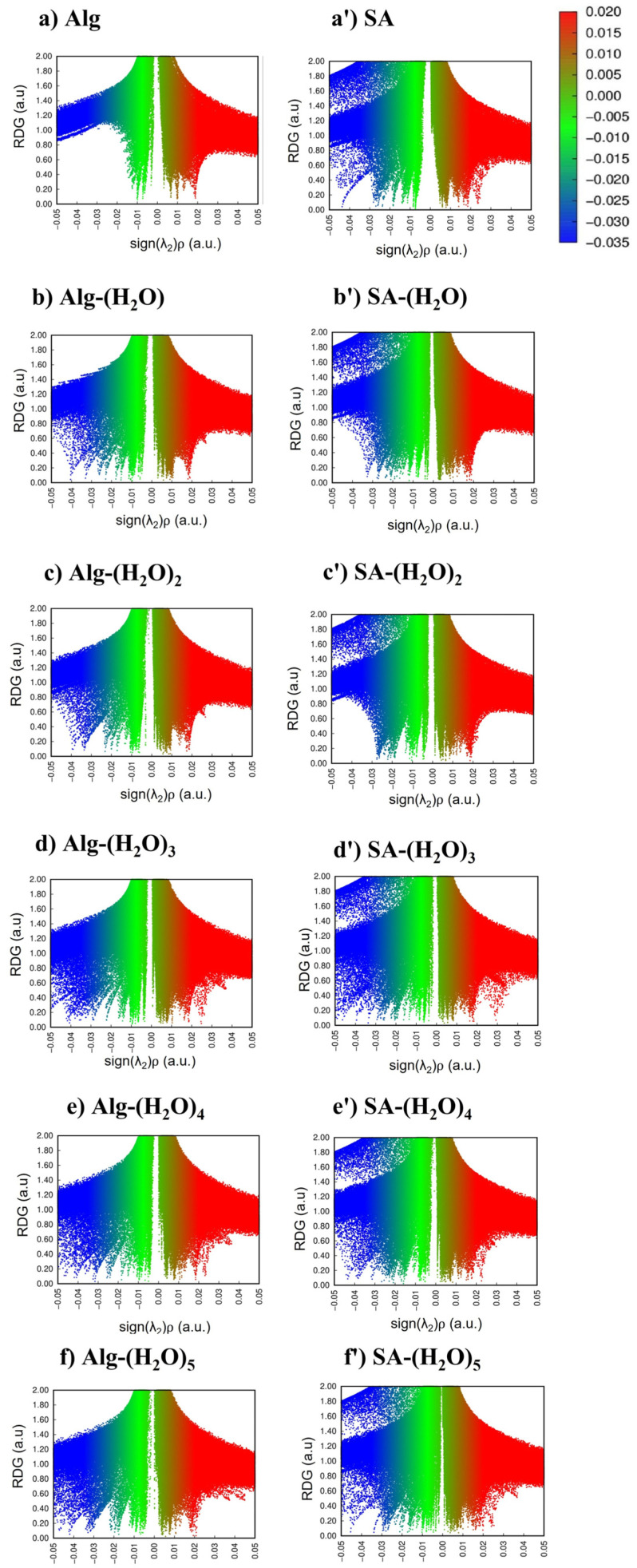
Scatter graph of RDGs for alg (**a**), alg-(H_2_O)_n_ complexes (**b**–**f**), SA (**a′**), and SA-H_2_O complexes (**b′**–**f′**).

The nature of the inter- and intra-molecular interaction of Alg-H_2_O complexes and SA-H_2_O complexes was examined by plotting the NCI index and RDG. By NCI-RDG analysis, Van der Walls interactions, hydrogen bonds, and steric effects were represented by the green, blue and red colors, respectively [46,48]). According to the 2D RDG graph of sign (λ_2_)ρ versus RDG value shown in Figure 2, on the horizontal axis, the RDG isosurface is 0.05 (with a range of −0.05 to 0.05). On the vertical axis, the density of all electrons’ (ρ) values ranged from −0.035 a.u to +0.02 a.u. The existing interaction could be characterized by the value of the secondary eigenvalue of the electron density Hessian matrix (λ_2_) and ρ. The strong attraction that refers to hydrogen bonds and halogen bonds has a value of λ_2_ < 0 and ρ > 0. Meanwhile, the interaction that acquires the value of λ_2_ ≈ 0 and ρ ≈0 is identified as a Van der Waals (VdW) interaction. Strong repulsion or steric effect in-rings and cages are defined by values of λ_2_ > 0 and ρ > 0 [27]. The sign of λ_2_ changes according to the function values of $\rho_{\left(r\right)};$ hence the amount sign(λ_2_)ρ acts as the new horizontal ordinate replacing ρ. It can be seen that the hydrogen bond area increased as more water molecules added to the Alg-(H_2_O)_n_ and SA-(H_2_O)_n_ complexes. The density of the SA-(H_2_O)_n_ complexes in the blue region is denser (more negative) than that of the Alg-(H_2_O)_n_ complexes. On the SA-(H_2_O)_n_ graph (Figure 2a′–f′), a strong metal-nonmetal interaction on Na…O is indicated by a blue color spike lying around −0.03 to −0.05 a.u. with a large RDG value. The phenomena of metal-nonmetal interactions on titanium and polyacrylamide (PAM) similarly exhibited an RDG value, with more spikes appearing in the attractive interactions area [48].

**Figure 3:**
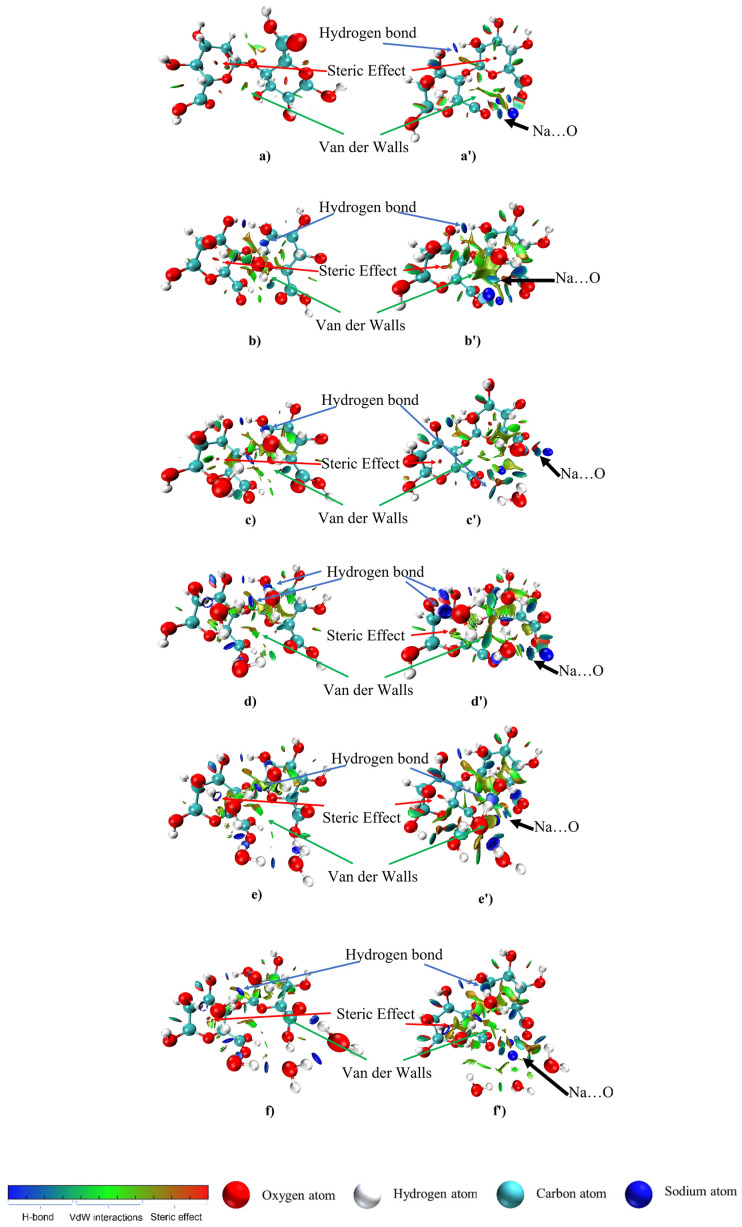
Three-dimensional RDG isosurface of (**a**) Alg, (**a′**) SA, (**a**–**f**) Alg-(H_2_O) complexes, and (**a′**–**f′**) SA-(H_2_O)_n_ complexes.

The visualizations of hydrogen bonds, the VdW interactions and steric effects were also presented with a three-dimensional (3D) RDG isosurface in Figure 3 for better understanding. Hydrogen bonds occurred between the water molecule and alginate and among the water molecules, which built the area of hydrogen bonds. Interestingly, the density of the steric effect site was also enhanced due to the new cyclic formation established by the hydrogen bonds between the water…water…alginate molecules. Cyclic formations on the complex structures were illustrated by the Alg-(H_2_O)_5_ structure (Figure S5). These results hypothetically proposed that the addition of water molecules will not extend the distance between the alginate and water molecule because of the attraction force with the hydrophile group from the alginate and other water molecules that could finally form a cyclic formation. Cyclic formation with water molecules also occurred in previous studies [21,49]. The Alg-(H_2_O)_n_ complexes showed hydrogen bonding (O…H), which is shown in blue. The strong metal-nonmetal interactions on SA-(H_2_O)_n_ complexes were also detected from the 3D RDG isosurface with blue color discs (Na…O). In addition, Van der Waals interactions in the SA-(H_2_O)_n_ complexes were more numerous than the Alg-(H_2_O)_n_ complexes, which was indicated by the green area being more numerous and broader. Therefore, this indicates that alginate has a decent water uptake, which could be investigated by the distance between the farthest water molecule and the closest alginate’s hydrophile group.

## 3. Conclusions

The interaction between alginic acid or sodium alginate and water molecules was evaluated using the DFT method and basis set of 6-31++G** through this in silico study. The results could support the idea of alginate as a hydrogel material as shown by the fact that the more water molecules that were added to the complex, the higher the interaction energy obtained became. NBO, AIM and RDG-NCI analyses were conducted to investigate the complexes’ intermolecular hydrogen bonds. This statement was confirmed by the AIM results as well. Furthermore, the increase in hydrogen bond interactions as the water molecules enhanced, which appeared in RDG and NCI analyses, also reinforced the results. This study concluded that alginate and water molecules interacted with each other through medium to strong hydrogen bond interactions, while sodium alginate and water molecules interacted through metal-nonmetal interactions.

## 4. Methods

### 4.1. Computational Analysis Using the Density Functional Theory (DFT-D3) Method

All calculations in this study were fulfilled by the density functional theory (DFT-D3) method using the hybrid of Becke’s three-parameter Lee, Yang, and Parr (B3LYP) functional and the basis set 6-31++G** [24,26]. A solvation model based on density (SMD) was used to calculate the effect of the water as a solvent [26,50]. The MM-alginate dimer structure was collected from PubChem (CID: 45048803). The optimization of each molecule was conducted to gain the minimum energies. After the most stable alginate molecule, as well as a water molecule, were found, both interacted together to obtain the complexes of Alg-(H_2_O)_n_ and SA-(H_2_O)_n_ minimum energy. In order to clarify the stability of those molecules and complexes, their frequencies were calculated. If the results are all positive, stability has been acquired.

### 4.2. Natural Bond Orbital (NBO)

NBO calculations were performed to estimate the interaction binding energy type, reactivity to transport proton, and charge distribution of the complexes [26,51]. The equation below defines the hyper conjugative interaction energy between an occupied (i) and an unoccupied (j), applying the theory of second-order perturbation.

$$E^{2}=\Delta E_{ij}=q_{i}\frac{F_{i.j}^{2}}{\varepsilon_{j}-\varepsilon_{i}}\;$$

The orbital stabilizing energy is indicated by the value of *E*^2^, where it is influenced by the energy of an NBO donor ($\varepsilon_{i}$) and the acceptor ($\varepsilon_{j}$), the occupancy of donating orbitals $(q_{i}$), as well as the NBO Fock matrix element between *i* and *j* orbitals ($F_{i.j}^{2}$) [20]. The NBO method could also investigate the intra- and intermolecular interactions by providing orbital electron density with the highest possible percentage that corresponds to a molecule’s Lewis structure precisely [27].

### 4.3. Quantum Theory Atom in Molecule (QTAIM) Analysis

QTAIM analysis was done by Multiwfn software, which calculates hydrogen bonding energy at the BCP index; its results are usually used to support the NBO analysis [27]. The theory of atoms-in-molecules also contemplates the nature of intermolecular hydrogen bonds that exist in the most stable complexes [21]. Valuable parameters such as the Laplacian and ellipticity are the results of QTAIM analysis. Ellipticity (ε) is known as the measure of a “double bond” or π character. The Laplacian ($\nabla^{2}\rho$) defines the local charge concentration or depletion and has the equation below as the parameters at the BCP;

$$\nabla^{2}\rho =\lambda_{1}+\lambda_{2}+\lambda_{3}$$

where $\lambda_{1}$, $\lambda_{2}$, and $\lambda_{3}$ represent the density at the critical point. The data were also made more accurate by electronic energy density (H_(BCP)_), which was calculated with the following equation;

H_(BCP)_ = G_(BCP)_ + V_(BCP)_

G_(BCP)_ represents the kinetic energy density, and V_(BCP)_ defines the potential energy density. With the result of $\nabla^{2}\;\rho$ and H_(BCP),_ the type of interaction could be predicted [52,53,54]. A weak covalent interaction, which means a strong electrostatic bond, is the result of $\nabla^{2}\rho$ (+) and H_(BCP)_ (+). A strong interaction or strong covalent bond is provided by $\nabla^{2}\rho$ (−) and H_(BCP)_ (−). A medium-strength or partially covalent bond is the result of $\nabla^{2}\rho$ (+) and H_(BCP)_ (−). The ratio of |V/G| was used as a reliable parameter to classify the different interactions. If |V/G| < 1, it refers to the weak interactions, while 1 < |V/G| < 2 refers to the medium interactions, and |V/G| > 2 refers to the strong interactions [27].

### 4.4. HOMO-LUMO Energy Analysis

A HOMO-LUMO energy analysis calculation was employed to evaluate the electron transfer reactivity of the molecule. The results were gathered from Multiwfn software [48,55].

### 4.5. Reduced Density Gradient (RDG) and Non-Covalent Interaction (NCI)

NCI-RDG analysis was conducted based on previous literature [56]. RDG-NCI analysis was used to identify the intramolecular interactions as well as the nature of the weak interactions that occur between water molecules and alginate. The information about non-covalent interactions, such as hydrogen bonds, tends to be provided by the NCI index, while the reduced density gradient (RDG) is determined by this following equation.

$$\mathrm{RDG}=\frac{1}{2{\left(3\pi^{2}\right)}^{1/3}}\frac{\left|\nabla \rho_{\left(r\right)}\right|}{\rho_{\left(r\right)}{}^{4/3}}$$

The complexes’ non-covalent interaction could be defined by the RDG value. The sign of (λ_2_)$\rho_{\left(r\right)}$ and NCI-RDG plots were derived from the Multiwfn program, and the RDG versus sign (λ_2_)$\rho_{\left(r\right)}$ of all complexes were displayed in scatter graphs.

## Figures and Tables

**Table 1:** Conformation and molecular volume value of complexes.

Complexes	χ	φ	ψ	Molecular Surface Volume (Å^3^)
Alg-(H_2_O)n				
0	48.964	−74.120	−112.542	367.23
1	158.751	45.423	−114.965	380.92
2	162.108	47.435	−116.299	404.53
3	152.732	34.373	−110.415	424.91
4	146.237	27.335	−108.499	447
5	143.925	24.970	−108.771	469
SA-(H_2_O)n				
0	160.468	47.475	−118.119	382.27
1	164.968	47.490	−116.093	405.12
2	179.733	76.410	−132.525	430.25
3	157.636	48.173	−121.836	445.81
4	152.083	35.460	−113.467	471.3
5	150.612	30.401	−107.584	500.1

**Table 2:** Bond length detail of alginate and alginate-(H_2_O)_n_ complexes.

Complex	Distance (Å)			
	(O11-H43)	(O7-H39)	(C25=O13)	(C17-O5)
Alg-(H_2_O)_n_				
0	0.9756	0.97005	1.21721	1.42388
1	0.97657	0.97968	1.22097	1.42452
2	0.97608	0.97118	1.2214	1.42434
3	0.976	1.00262	1.22167	1.4255
4	1.00498	1.00171	1.22795	1.42574
5	1.02644	1.00607	1.23184	1.42676
SA-(H_2_O)_n_	(O12…Na *)	(O11…Na *)	(C25-O13)	(C24-O10)
0	2.1609	2.1965	1.2507	1.2555
1	2.1888	2.1820	1.2645	1.2503
2	2.3307	2.2307	1.2882	1.2566
3	2.2377	2.3434	1.2583	1.2541
4	2.2509	2.1683	1.2671	1.2477
5	2.2136	2.1948	1.2648	1.2596

* The closest Na with carboxylate group.

**Table 3:** The chemical descriptors of the system.

Complexes	E_HOMO_ (eV)	E_LUMO_ (eV)	ΔE (eV)
Alg-(H_2_O)_n_			
0	−7.19	−0.35	6.84
1	−6.96	−0.52	6.43
2	−6.98	−0.56	6.42
3	−6.99	−0.59	6.39
4	−6.96	−0.67	6.29
5	−6.95	−0.61	6.33
SA-(H_2_O)_n_			
0	−6.89	−0.46	6.43
1	−6.98	−0.36	6.59
2	−6.96	−0.64	6.31
3	−6.86	−0.44	6.42
4	−6.89	−0.39	6.51
5	−6.77	−0.56	6.21

## Data Availability

Not applicable.

## Supplementary Materials

The following supporting information can be downloaded at: https://www.mdpi.com/article/10.3390/gels8110703/s1, Figure S1: Optimized structure and molecular electrostatic potential of water (**a** and **b**, respectively); Figure S2: Conformation of optimized SA and Alg dimers structures. Water molecules and sodium ions were precluded on complexes for facilitating the visualization; Figure S3: Optimized structure of Alg-(H_2_O)_n=1–5_ and SA-(H_2_O)_n=1-5_ complexes; Figure S4: The molecular graphs of Alg-(H_2_O)_n_ complexes (**a**–**e**) and SA-(H_2_O)_n_ complexes (**a′**–**e′**). The BCP are represented by the unadorned orange sphere and the red circle indicates the presence of water molecule; Figure S5: Optimized structure of Alg-(H_2_O)_5_ (**a**) and cyclic on Alg-(H_2_O) structure (**b**–**d**); Table S1: Dipole moment; Table S2: Deformation energy; Table S3: Selected intermolecular acceptor-donor interactions and second-order perturbation stabilization energies of the H-bonded complexes in alginic acid-water; Table S4: Selected intermolecular acceptor-donor interactions and second-order perturbation stabilization energies of the Na-bonded complexes in sodium alginate-water; Table S5: The analysis of the bond critical points of the different Alg-water complexes by QTAIM; Table S6: The analysis of the bond critical points of the different SA-water complexes by QTAIM.

## References

[1] Li Y., Huang G., Zhang X., Li B., Chen Y., Lu T., Lu T.J., Xu F. (2013). Magnetic Hydrogels and Their Potential Biomedical Applications. Adv. Funct. Mater..

[2] Abou-Yousef H., Dacrory S., Hasanin M., Saber E., Kamel S. (2021). Biocompatible Hydrogel Based on Aldehyde-Functionalized Cellulose and Chitosan for Potential Control Drug Release. Sustain. Chem. Pharm..

[3] Bashir S., Hina M., Iqbal J., Rajpar A.H., Mujtaba M.A., Alghamdi N.A., Wageh S., Ramesh K., Ramesh S. (2020). Fundamental Concepts of Hydrogels: Synthesis, Properties, and Their Applications. Polymers.

[4] Ahmed E.M. (2015). Hydrogel: Preparation, Characterization, and Applications: A Review. J. Adv. Res..

[5] Abasalizadeh F., Moghaddam S.V., Alizadeh E., Akbari E., Kashani E., Fazljou S.M.B., Torbati M., Akbarzadeh A. (2020). Alginate-Based Hydrogels as Drug Delivery Vehicles in Cancer Treatment and Their Applications in Wound Dressing and 3D Bioprinting. J. Biol. Eng..

[6] Zhang H., Cheng J., Ao Q. (2021). Preparation of Alginate-Based Biomaterials and Their Applications in Biomedicine. Mar. Drugs.

[7] Ma G., Ran F., Yang Q., Feng E., Lei Z. (2015). Eco-Friendly Superabsorbent Composite Based on Sodium Alginate and Organo-Loess with High Swelling Properties. RSC Adv..

[8] Pawar S.N., Edgar K.J. (2012). Alginate Derivatization: A Review of Chemistry, Properties and Applications. Biomaterials.

[9] Jalababu R., Veni S.S., Reddy K.V.N.S. (2018). Synthesis and Characterization of Dual Responsive Sodium Alginate-g-Acryloyl Phenylalanine-Poly N-Isopropyl Acrylamide Smart Hydrogels for the Controlled Release of Anticancer Drug. J. Drug Deliv. Sci. Technol..

[10] Lima D.S., Tenório-Neto E.T., Lima-Tenório M.K., Guilherme M.R., Scariot D.B., Nakamura C.V., Muniz E.C., Rubira A.F. (2018). PH-Responsive Alginate-Based Hydrogels for Protein Delivery. J. Mol. Liq..

[11] Anugrah D.S.B., Ramesh K., Kim M., Hyun K., Lim K.T. (2019). Near-Infrared Light-Responsive Alginate Hydrogels Based on Diselenide-Containing Cross-Linkage for on Demand Degradation and Drug Release. Carbohydr. Polym..

[12] Siboro S.A.P., Anugrah D.S.B., Ramesh K., Park S.-H., Kim H.-R., Lim K.T. (2021). Tunable Porosity of Covalently Crosslinked Alginate-Based Hydrogels and Its Significance in Drug Release Behavior. Carbohydr. Polym..

[13] Kang J.I., Park K.M., Park K.D. (2019). Oxygen-Generating Alginate Hydrogels as a Bioactive Acellular Matrix for Facilitating Wound Healing. J. Ind. Eng. Chem..

[14] Pan H., Zhang C., Wang T., Chen J., Sun S.K. (2019). In Situ Fabrication of Intelligent Photothermal Indocyanine Green-Alginate Hydrogel for Localized Tumor Ablation. ACS Appl. Mater. Interfaces.

[15] Urzedo A.L., Gonçalves M.C., Nascimento M.H.M., Lombello C.B., Nakazato G., Seabra A.B. (2020). Cytotoxicity and Antibacterial Activity of Alginate Hydrogel Containing Nitric Oxide Donor and Silver Nanoparticles for Topical Applications. ACS Biomater. Sci. Eng..

[16] Bekri L., Zouaoui-Rabah M., Springborg M., Rahal M.S. (2018). A Structural DFT Study of MM, GG, MG, and GM Alginic Acid Disaccharides and Reactivity of the MG Metallic Complexes. J. Mol. Model..

[17] Sanchez-Ballester N.M., Bataille B., Soulairol I. (2021). Sodium Alginate and Alginic Acid as Pharmaceutical Excipients for Tablet Formulation: Structure-Function Relationship. Carbohydr. Polym..

[18] Li Z.J., Srebnik S., Rojas O.J. (2022). Competing Effects of Hydration and Cation Complexation in Single-Chain Alginate. Biomacromolecules.

[19] Ardiles C.S., Rodríguez C.C. (2021). Theoretical Study for Determining the Type of Interactions between a GG Block of an Alginate Chain with Metals Cu2+, Mn2+, Ca2+ and Mg2+. Arab. J. Chem..

[20] Singla N., Chowdhury P. (2013). Density Functional Investigation of Photo Induced Intramolecular Proton Transfer (IPT) in Indole-7-Carboxaldehyde and Its Experimental Verification. J. Mol. Struct..

[21] Hammami F., Ghalla H., Nasr S. (2015). Intermolecular Hydrogen Bonds in Urea-Water Complexes: DFT, NBO, and AIM Analysis. Comput. Theor. Chem..

[22] Rahmawati S., Radiman C.L., Martoprawiro M.A. (2018). Density Functional Theory (DFT) and Natural Bond Orbital (NBO) Analysis of Intermolecular Hydrogen Bond Interaction in “Phosphorylated Nata de Coco-Water”. Indones. J. Chem..

[23] Valiev M., Bylaska E.J., Govind N., Kowalski K., Straatsma T.P., Van Dam H.J.J., Wang D., Nieplocha J., Apra E., Windus T.L. (2010). NWChem: A Comprehensive and Scalable Open-Source Solution for Large Scale Molecular Simulations. Comput. Phys. Commun..

[24] Anugrah D.S.B., Darmalim L.V., Putro P.A., Nuratikah L.D., Sasongko N.A., Siahaan P., Yulandi A. (2021). Computational Evaluation of Intermolecular Interaction in Poly(Styrene-Maleic Acid)-Water Complexes Using Density Functional Theory. Indones. J. Chem..

[25] Cisneros G.A., Wikfeldt K.T., Ojamäe L., Lu J., Xu Y., Torabifard H., Bartók A.P., Csányi G., Molinero V., Paesani F. (2016). Modeling Molecular Interactions in Water: From Pairwise to Many-Body Potential Energy Functions. Chem. Rev..

[26] Pontoh R., Rarisavitri V.E., Yang C.C., Putra M.F., Anugrah D.S.B. (2022). Density Functional Theory Study of Intermolecular Interactions between Amylum and Cellulose. Indones. J. Chem..

[27] Siahaan P., Sasongko N.A., Lusiana R.A., Prasasty V.D., Martoprawiro M.A. (2021). The Validation of Molecular Interaction among Dimer Chitosan with Urea and Creatinine Using Density Functional Theory: In Application for Hemodyalisis Membrane. Int. J. Biol. Macromol..

[28] Cortes E., Márquez E., Mora J.R., Puello E., Rangel N., De Moya A., Trilleras J. (2019). Theoretical Study of the Adsorption Process of Antimalarial Drugs into Acrylamide-Base Hydrogel Model Using DFT Methods: The First Approach to the Rational Design of a Controlled Drug Delivery System. Processes.

[29] Uto T., Yui T. (2018). DFT Optimization of Isolated Molecular Chain Sheet Models Constituting Native Cellulose Crystal Structures. ACS Omega.

[30] Aravamudhan A., Ramos D.M., Nada A.A., Kumbar S.G., Kumbar S.G., Laurencin C.T., Deng M. (2014). Natural Polymers. Natural and Synthetic Biomedical Polymers.

[31] Kania D., Yunus R., Omar R., Abdul S., Mohamed B., Aulia A. (2021). Journal of Petroleum Science and Engineering Lubricity Performance of Non-Ionic Surfactants in High-Solid Drilling Fluids : A Perspective from Quantum Chemical Calculations and Filtration Properties. J. Pet. Sci. Eng..

[32] Costa M.P.M., Prates L.M., Baptista L., Cruz M.T.M., Ferreira I.L.M. (2018). Interaction of Polyelectrolyte Complex between Sodium Alginate and Chitosan Dimers with a Single Glyphosate Molecule: A DFT and NBO Study. Carbohydr. Polym..

[33] Koushesh Saba M., Amini R., Acevedo-Fani A., Soliva-Fortuny R., Martín-Belloso O., Sharaf Eddin A., Ibrahim S.A., Tahergorabi R., Dissertation D., Sun Q. (2019). Edible Films/Coating with Tailored Properties for Active Packaging of Meat, Fish and Derived Products. Curr. Opin. Food Sci..

[34] Namviriyachote N., Lipipun V., Akkhawattanangkul Y., Charoonrut P., Ritthidej G.C. (2019). Development of Polyurethane Foam Dressing Containing Silver and Asiaticoside for Healing of Dermal Wound. Asian J. Pharm. Sci..

[35] Baghbani F., Chegeni M., Moztarzadeh F., Mohandesi J.A., Mokhtari-Dizaji M. (2017). Ultrasonic Nanotherapy of Breast Cancer Using Novel Ultrasound-Responsive Alginate-Shelled Perfluorohexane Nanodroplets: In Vitro and in Vivo Evaluation. Mater. Sci. Eng. C.

[36] French A.D. (2015). Energy Maps for Glycosidic Linkage Conformations. Methods Mol. Biol..

[37] Brus J., Urbanova M., Czernek J., Pavelkova M., Kubova K., Vyslouzil J., Abbrent S., Konefal R., Horský J., Vetchy D. (2017). Structure and Dynamics of Alginate Gels Cross-Linked by Polyvalent Ions Probed via Solid State NMR Spectroscopy. Biomacromolecules.

[38] Agulhon P., Markova V., Robitzer M., Quignard F., Mineva T. (2012). Structure of Alginate Gels: Interaction of Diuronate Units with Divalent Cations from Density Functional Calculations. Biomacromolecules.

[39] Guo X., Wang Y., Qin Y., Shen P., Peng Q. (2020). Structures, Properties and Application of Alginic Acid: A Review. Int. J. Biol. Macromol..

[40] García-Astrain C., Avérous L. (2018). Synthesis and Evaluation of Functional Alginate Hydrogels Based on Click Chemistry for Drug Delivery Applications. Carbohydr. Polym..

[41] Tamukong P.K., Khait Y.G., Hoffmann M.R. (2017). Accurate Dissociation of Chemical Bonds Using DFT-in-DFT Embedding Theory with External Orbital Orthogonality. J. Phys. Chem. A.

[42] Rahmawati S., Radiman C.L., Martoprawiro M.A. (2017). Ab Initio Study of Proton Transfer and Hydration on Phosphorylated Nata de Coco. Indones. J. Chem..

[43] Chuang J.-J., Huang Y.-Y., Lo S.-H., Hsu T.-F., Huang W.-Y., Huang S.-L., Lin Y.-S. (2017). Effects of PH on the Shape of Alginate Particles and Its Release Behavior. Int. J. Polym. Sci..

[44] Szekalska M., Puciłowska A., Szymańska E., Ciosek P., Winnicka K. (2016). Alginate: Current Use and Future Perspectives in Pharmaceutical and Biomedical Applications. Int. J. Polym. Sci..

[45] Lee K.Y., Mooney D.J. (2012). Alginate: Properties and Biomedical Applications. Prog. Polym. Sci..

[46] Venkataramanan N.S., Suvitha A., Kawazoe Y. (2017). Intermolecular Interaction in Nucleobases and Dimethyl Sulfoxide/Water Molecules: A DFT, NBO, AIM and NCI Analysis. J. Mol. Graph. Model..

[47] Safia H., Ismahan L., Abdelkrim G., Mouna C., Leila N., Fatiha M. (2019). Density Functional Theories Study of the Interactions between Host β-Cyclodextrin and Guest 8-Anilinonaphthalene-1-Sulfonate: Molecular Structure, HOMO, LUMO, NBO, QTAIM and NMR Analyses. J. Mol. Liq..

[48] Awasthi S., Gaur J.K., Pandey S.K., Bobji M.S., Srivastava C. (2021). High-Strength, Strongly Bonded Nanocomposite Hydrogels for Cartilage Repair. ACS Appl. Mater. Interfaces.

[49] Akman F., Issaoui N., Kazachenko A.S. (2020). Intermolecular Hydrogen Bond Interactions in the Thiourea/Water Complexes (Thio-(H2O)n) (n = 1, …, 5): X-Ray, DFT, NBO, AIM, and RDG Analyses. J. Mol. Model..

[50] Ehrlich S., Moellmann J., Grimme S. (2013). Dispersion-Corrected Density Functional Theory for Aromatic Interactions in Complex Systems. Acc. Chem. Res..

[51] Glendening E.D., Landis C.R., Weinhold F. (2019). NBO 7.0: New Vistas in Localized and Delocalized Chemical Bonding Theory. J. Comput. Chem..

[52] Grabowski S.J. (2004). Hydrogen Bonding Strength-Measures Based on Geometric and Topological Parameters. J. Phys. Org. Chem..

[53] Rozas I., Alkorta I., Elguero J. (2000). Behavior of Ylides Containing N, O, and C Atoms as Hydrogen Bond Acceptors. J. Am. Chem. Soc..

[54] Lane J.R., Contreras-García J., Piquemal J.P., Miller B.J., Kjaergaard H.G. (2013). Are Bond Critical Points Really Critical for Hydrogen Bonding?. J. Chem. Theory Comput..

[55] Lu T., Chen F. (2012). Multiwfn: A Multifunctional Wavefunction Analyzer. J. Comput. Chem..

[56] Johnson E.R., Keinan S., Mori-Sánchez P., Contreras-García J., Cohen A.J., Yang W. (2010). Revealing Noncovalent Interactions. J. Am. Chem. Soc..

