# Supplementary material for: Quantum Chemical Calculation for Intermolecular Interactions of Alginate Dimer-Water Molecules
Source: Gels. 2022 Oct 31;8(11):703. doi: 10.3390/gels8110703 (PMC9689446; doi:10.3390/gels8110703)
Supplement: Supplementary file 1 [file gels-08-00703-s001.zip › gels-1958724-supplementary.pdf]

Supporting Information

Quantum Chemical Calculation for Intermolecular Interactions of Alginate Dimer-Water Molecules

Daru Seto Bagus Anugrah [1,*], Laura Virdy Darmalim [1], Muhammad Rifky Irwanto Polanen [2], Permono Adi Putro [3], Nurwarrohman Andre Sasongko [4,5], Parsaoran Siahaan [4] and Zeno Rizqi Ramadhan [6]

[1] Biotechnology Study Program, Faculty of Biotechnology, Atma Jaya Catholic University of Indonesia,
BSD Campus, Tangerang 15345, Indonesia
[2] Food Technology Study Program, Faculty of Biotechnology, Atma Jaya Catholic University of Indonesia, BSD Campus, Tangerang 15345, Indonesia
[3] Department of Physics, Faculty of Science, Universitas Mandiri, Subang 41211, Indonesia
[4] Department of Chemistry, Faculty of Science and Mathematics, Diponegoro University, Semarang 50275, Indonesia
[5] Department of Chemistry, Pukyong National University, Busan 48513, Republic of Korea
[6] School of Chemistry, University of New South Wales, Sydney, NSW 2052, Australia
* Correspondence: darufile@gmail.com or daru.seto@atmajaya.ac.id

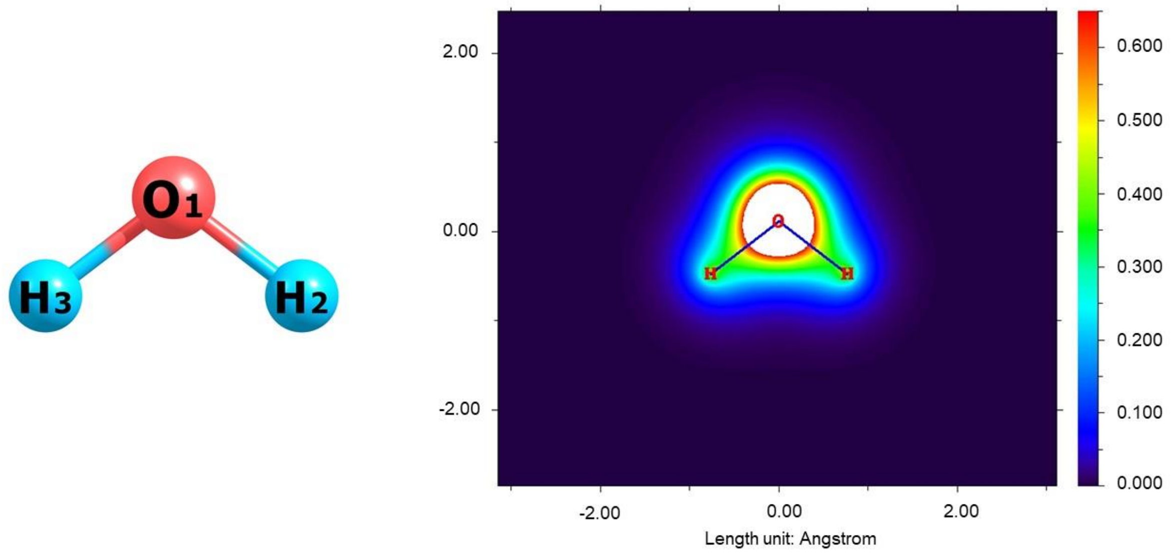

a) b)

Figure S1. Optimized structure and molecular electrostatic potential of water (**a** and **b**, respectively).

Table S1. Dipole moment

Structure	Total dipole moment (Debye)
Alg	4.03
SA	6.83

Table S2. Deformation energy

Structure	Energy (kcal/mol)		
	E_{opt}	$E_{isolated}$	E_{def}
Alg-$(H_2O)_1$	-955444.5		
SA-$(H_2O)_1$	-1158411.9		
Alg	-907460.9	-907484.9	24.1
SA	-1110433.7	-1110463.2	29.5

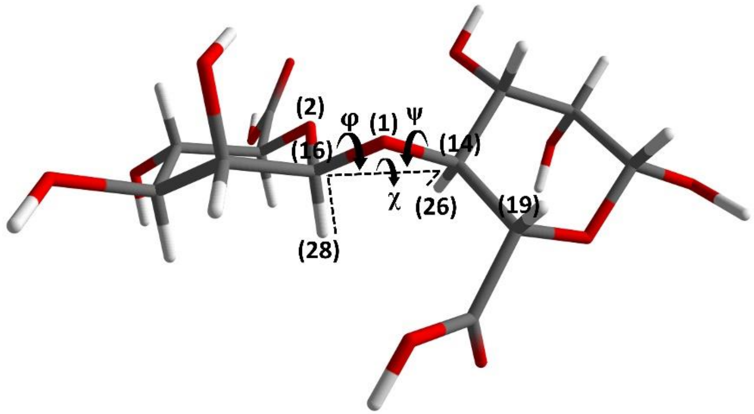

Alg

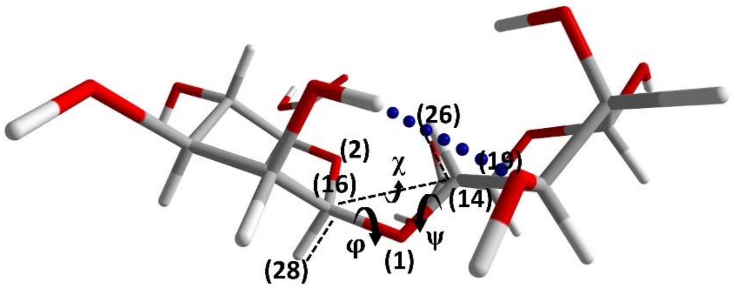

Alg-$(H_2O)_1$

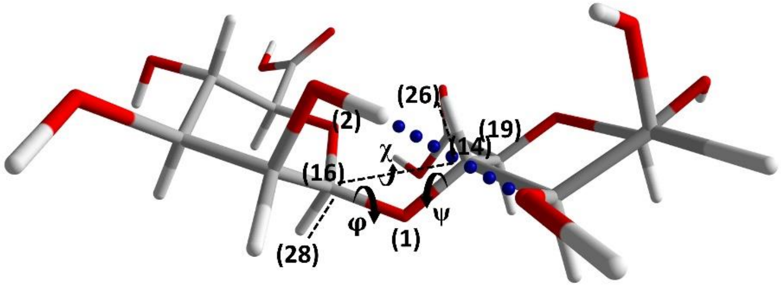

Alg-$(H_2O)_2$

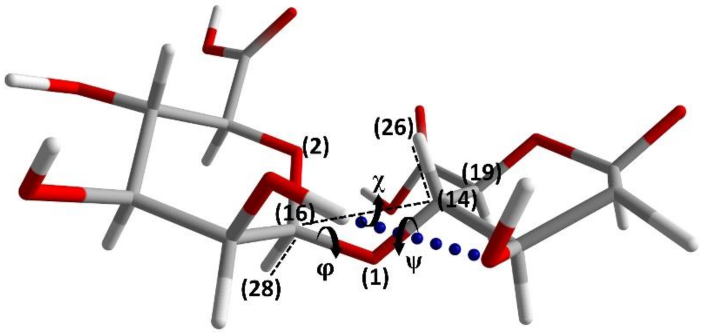

Alg-$(H_2O)_3$

χ = (28)-(16)-(14)-(26), φ = (2)-(16)-(1)-(14), ψ = (19)-(14)-(1)-(16)

Figure S2. Conformation of optimized SA and Alg dimers structures. Water molecules and sodium ions were precluded on complexes for facilitating the visualization.

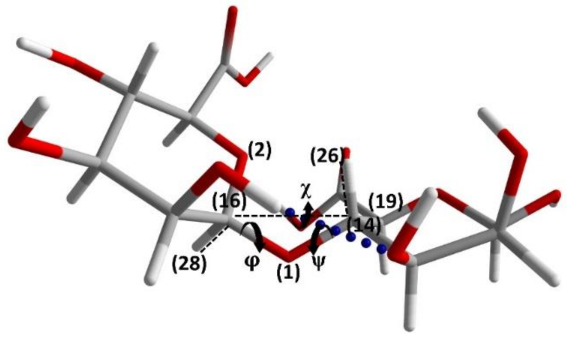

Alg-$(H_2O)_4$

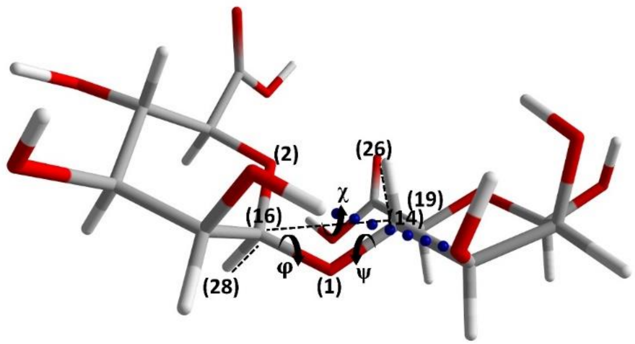

Alg-$(H_2O)_5$

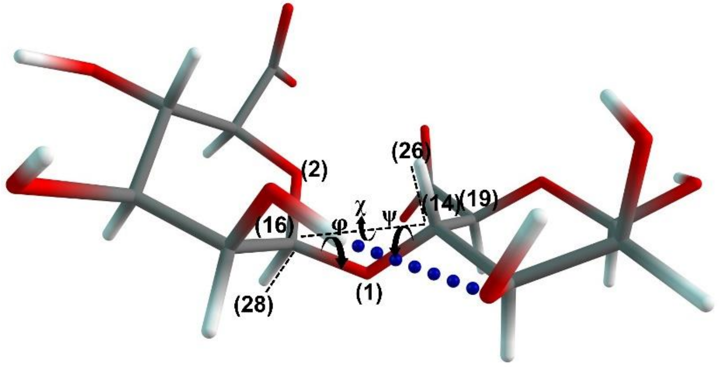

SA

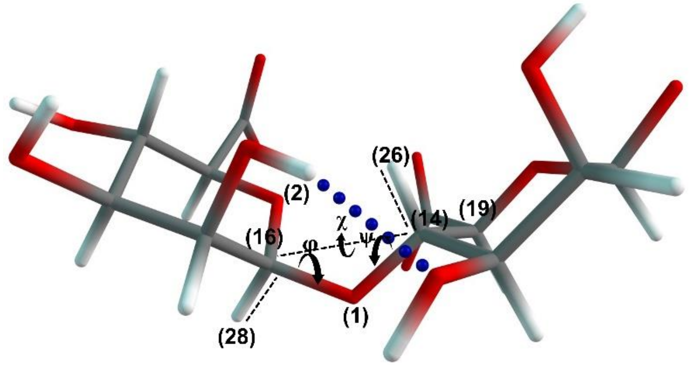

SA-$(H_2O)_1$

χ = (28)-(16)-(14)-(26), φ = (2)-(16)-(1)-(14), ψ = (19)-(14)-(1)-(16)

Figure S2. (*Continued*)

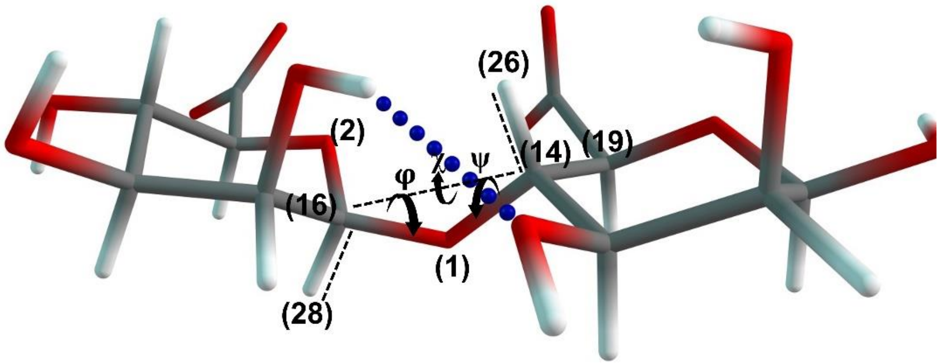

SA-$(H_2O)_2$

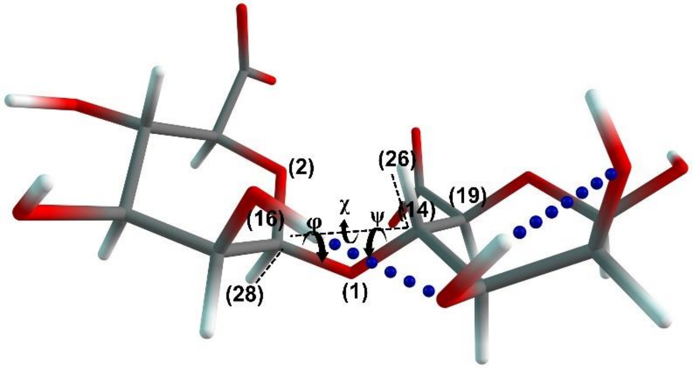

SA-$(H_2O)_3$

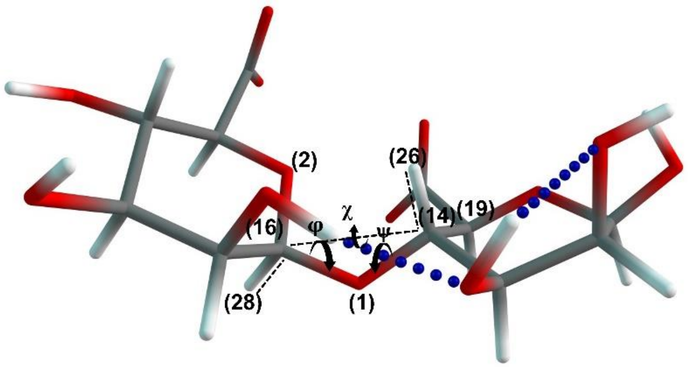

SA-$(H_2O)_4$

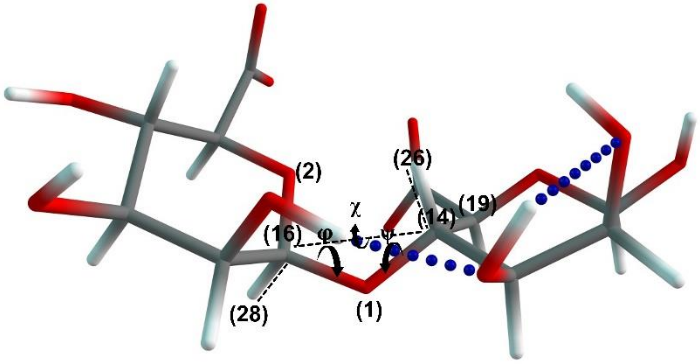

SA-$(H_2O)_5$

χ = (28)-(16)-(14)-(26), φ = (2)-(16)-(1)-(14), ψ = (19)-(14)-(1)-(16)

Figure S2. (*Continued*)

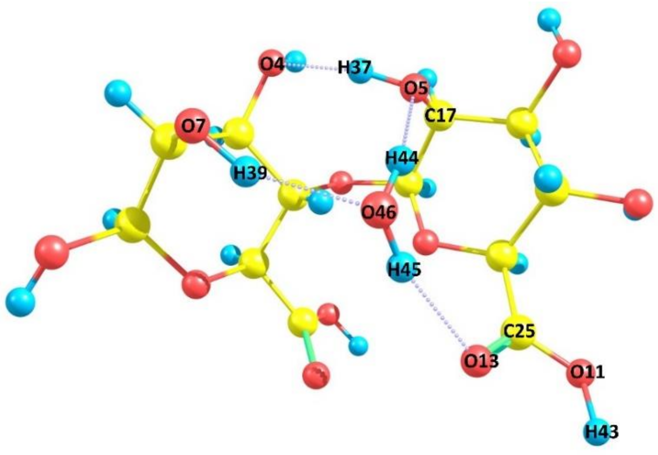

Alg-$(H_2O)_1$

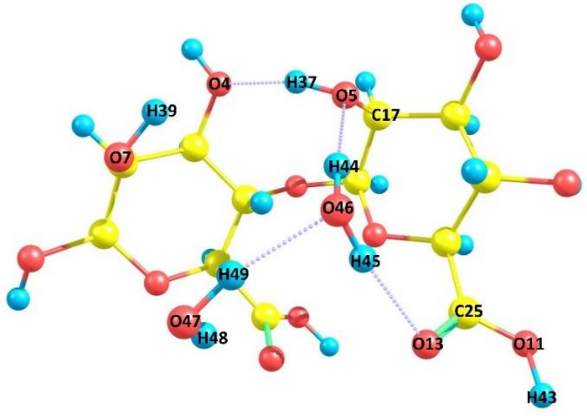

Alg-$(H_2O)_2$

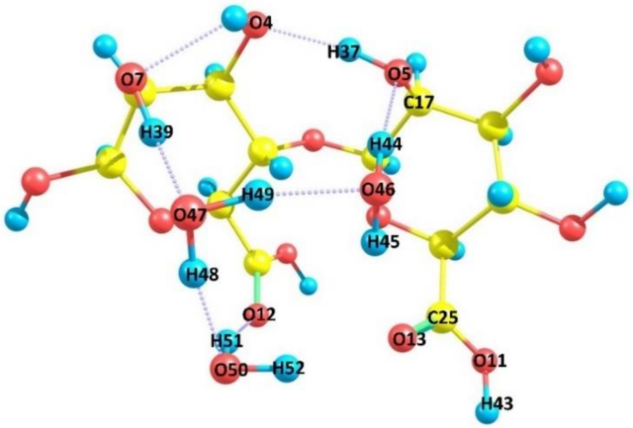

Alg-$(H_2O)_3$

Figure S3. Optimized structure of Alg-$(H_2O)_{n=1-5}$ and SA-$(H_2O)_{n=1-5}$ complexes.

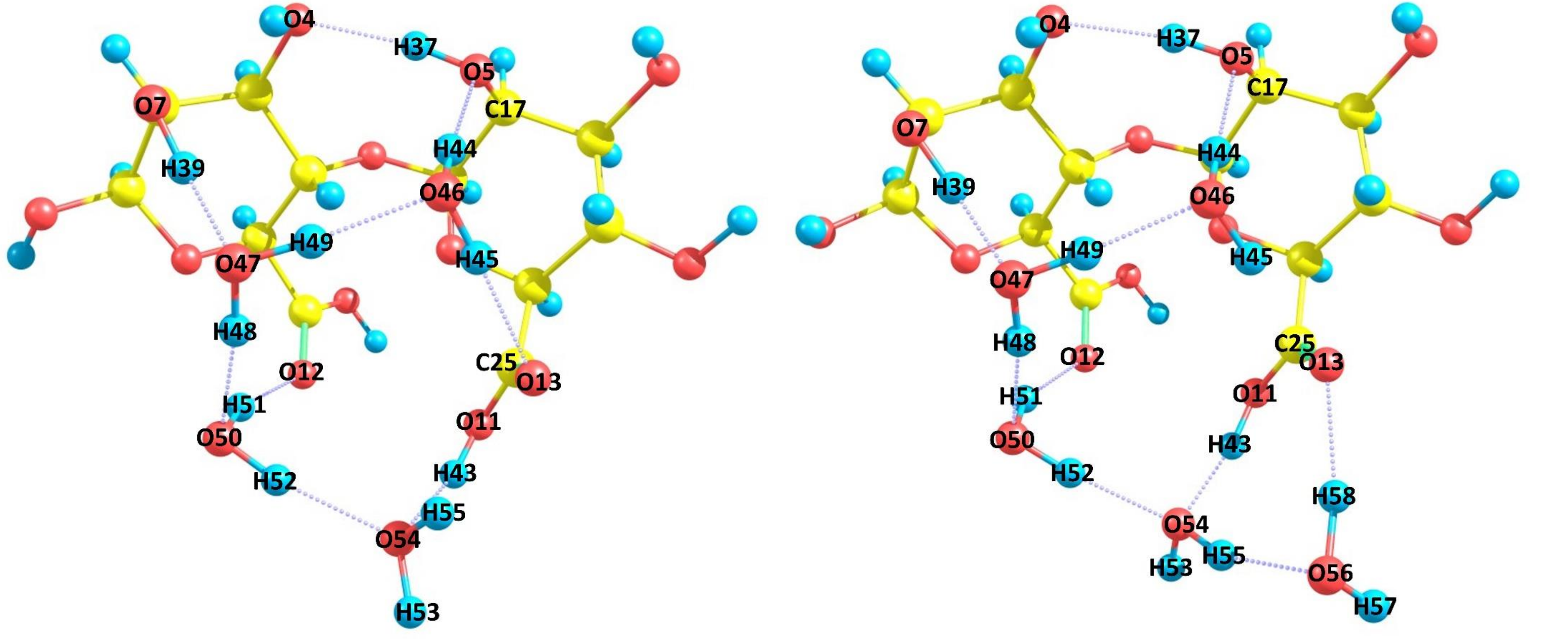

Alg-$(H_2O)_4$ Alg-$(H_2O)_5$

Figure S3. (*Continued*)

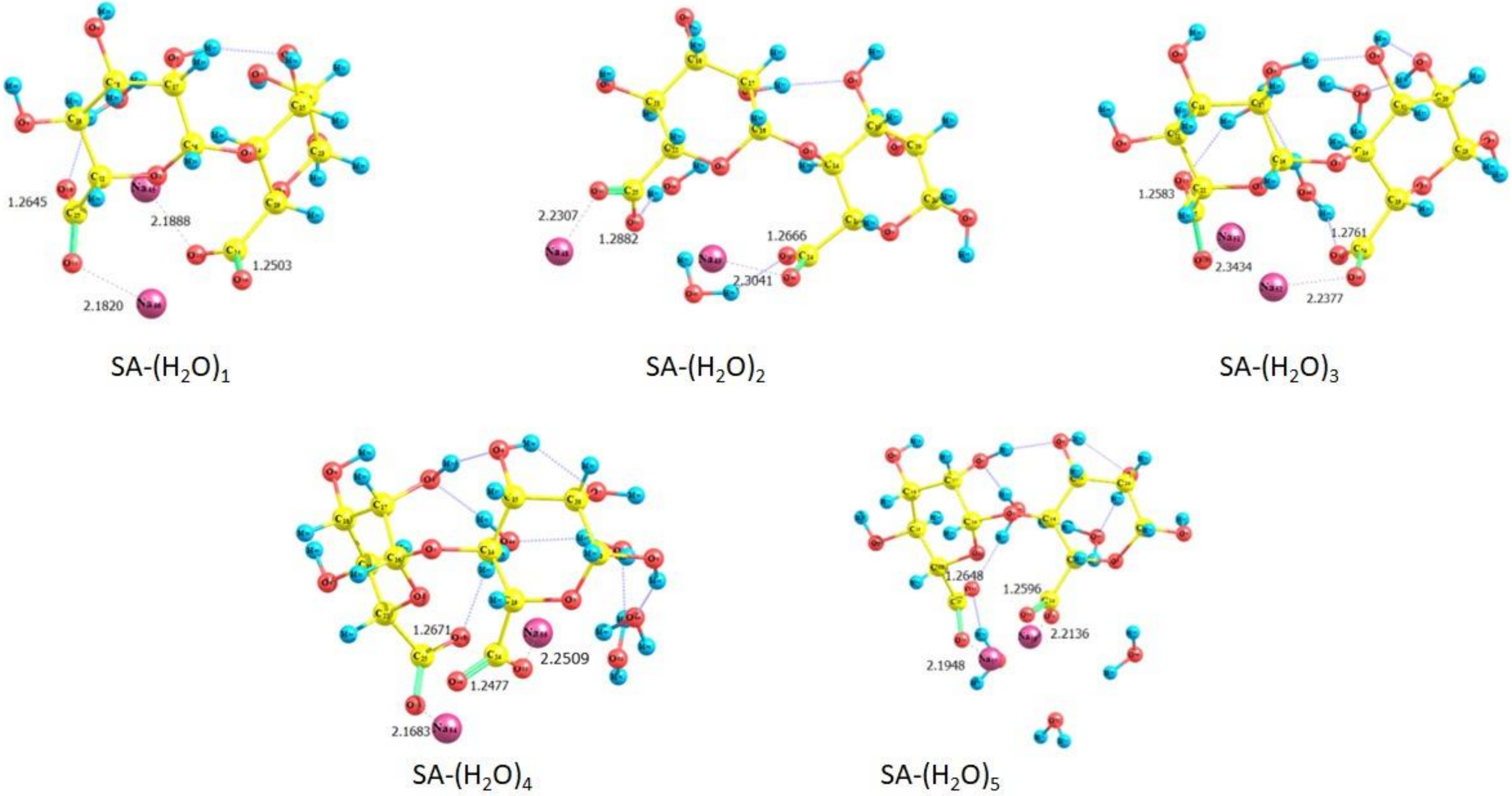

Figure S3. (*Continued*)

Table S3. Selected intermolecular acceptor-donor interactions and second-order perturbation stabilization energies of the H-bonded complexes in alginic acid-water.

Donor (i)	Acceptor (j)	$E^{(2)}$ (kcal/mol)	E(i)-E(j) (a.u)	F(i,j) (a.u)
Alg−H_2O				
LP (1) O5	BD*(1) H44-O46	3.82	0.98	0.055
LP (2) O5	BD*(1) H44-O46	13.19	0.85	0.094
LP (1) O13	BD*(1) H45-O46	3.86	1.09	0.058
LP (2) O13	BD*(1) H45-O46	5.88	0.80	0.061
LP (2) O46	BD*(1) O7-H39	11.89	0.80	0.087
Alg−$(H_2O)_2$				
LP (1) O5	BD*(1) H44-O46	4.06	0.99	0.057
LP (2) O5	BD*(1) H44-O46	13.70	0.87	0.097
LP (1) O13	BD*(1) H45-O46	3.42	1.09	0.055
LP (2) O13	BD*(1) H45-O46	6.01	0.80	0.062
LP (1) O12	BD*(1) O47-H48	2.47	1.13	0.047
LP (2) O12	BD*(1) O47-H48	4.78	0.84	0.056
LP (2) O46	BD*(1) O47-H49	16.08	0.87	0.106
Alg−$(H_2O)_3$				
LP (1) O5	BD*(1) H44-O46	3.10	0.96	0.049
LP (2) O5	BD*(1) H44-O46	16.73	0.85	0.106
LP (1) O13	BD*(1) H45-O46	2.98	1.08	0.051
LP (2) O13	BD*(1) H45-O46	6.15	0.79	0.062
LP (1) O12	BD*(1) O50-H51	7.45	1.08	0.080
LP (2) O12	BD*(1) O50-H51	8.85	0.80	0.075
LP (1) O13	BD*(1) O50-H52	3.67	1.09	0.056
LP (2) O46	BD*(1) O47-H49	23.31	0.85	0.125
LP (2) O47	BD*(1) O7-H39	37.88	0.82	0.157
LP (2) O50	BD*(1) O47-H48	23.29	0.85	0.126
Alg−$(H_2O)_4$				
LP (1) O5	BD*(1) H44-O46	2.87	0.82	0.011
LP (2) O5	BD*(1) H44-O46	14.39	0.84	0.098
LP (1) O13	BD*(1) H45-O46	3.97	1.09	0.059
LP (2) O13	BD*(1) H45-O46	3.15	0.79	0.045
LP (1) O12	BD*(1) O50-H51	7.22	1.09	0.079
LP (2) O12	BD*(1) O50-H51	9.09	0.80	0.076
LP (1) O13	BD*(1) O54-H55	2.42	0.72	0.037
LP (2) O46	BD*(1) O47-H49	23.65	0.87	0.128
LP (2) O47	BD*(1) O7-H39	36.55	0.83	0.156
LP (2) O50	BD*(1) O47-H48	24.08	0.85	0.128
LP (2) O54	BD*(1) O11-H43	28.57	0.85	0.139
LP (1) O54	BD*(1) O50-H52	14.15	1.01	0.107
Alg−$(H_2O)_5$				
LP (1) O5	BD*(1) H44-O46	2.71	0.97	0.046
LP (2) O5	BD*(1) H44-O46	14.13	0.84	0.097

LP (1) O13	BD*(1) H45-O46	2.14	1.09	0.043
LP (1) O12	BD*(1) O50-H51	6.42	1.09	0.075
LP (2) O12	BD*(1) O50-H51	8.61	0.80	0.074
LP (1) O13	BD*(1) O56-H58	7.22	1.04	0.077
LP (2) O13	BD*(1) O56-H58	10.90	0.75	0.081
LP (2) O46	BD*(1) O47-H49	24.73	0.88	0.132
LP (2) O47	BD*(1) O7-H39	39.43	0.82	0.161
LP (2) O50	BD*(1) O47-H48	24.90	0.85	0.130
LP (2) O54	BD*(1) O11-H43	46.21	0.83	0.175
LP (1) O54	BD*(1) O50-H52	15.52	0.98	0.110
LP (2) O56	BD*(1) O54-H55	28.93	0.79	0.135

Table S4. Selected intermolecular acceptor-donor interactions and second-order perturbation stabilization energies of the Na-bonded complexes in sodium alginate-water.

**Donor (i)**	**Acceptor (j)**	$E^{(2)}$ **(kcal/mol)**	E**(i)-**E**(j) (a.u)**	F**(i,j) (a.u)**
SA-$(H_2O)_1$				
CR (1) O 12	RY (8)Na 45	7.57	19.36	0.342
CR (1) O 12	RY (9)Na 45	9.64	19.67	0.389
CR (1) O 12	RY (12)Na 45	50.99	19.48	0.89
LP (1) O 12	RY (8)Na 45	13.75	0.82	0.095
LP (1) O 12	RY (9)Na 45	11.1	1.12	0.1
LP (1) O 12	RY (10)Na 45	4.38	1.12	0.063
LP (1) O 12	RY (12)Na 45	77.28	0.93	0.24
BD (1) O 12- C 24	RY (12)Na 45	33.02	1.2	0.178
CR (1) O 10	RY (5)Na 46	5.01	19.59	0.28
CR (1) O 10	RY (6)Na 46	9.76	19.47	0.389
CR (1) O 10	RY (10)Na 46	11.35	19.46	0.42
CR (1) O 11	RY (6)Na 46	13.67	19.46	0.46
CR (1) O 11	RY (10)Na 46	8.54	19.45	0.364
CR (1) O 11	RY (11)Na 46	27.67	19.33	0.653
CR (1) O 11	RY (12)Na 46	6.05	19.82	0.309
CR (1) O 12	RY (6)Na 46	7.16	19.47	0.333
CR (1) O 12	RY (7)Na 46	3.94	19.37	0.247
CR (1) O 12	RY (11)Na 46	28.58	19.35	0.664
CR (1) C 24	RY (11)Na 46	7.88	10.52	0.257
LP (1) O 10	RY (6)Na 46	13.91	0.9	0.1
LP (1) O 10	RY (10)Na 46	12.67	0.89	0.095
LP (1) O 11	RY (6)Na 46	17.34	0.9	0.112
LP (1) O 12	RY (11)Na 46	42.23	0.8	0.164
BD (1) O 11- C 25	RY (6)Na 46	11.24	1.19	0.103
BD (1) O 11- C 25	RY (10)Na 46	18.05	1.19	0.131
BD (1) O 11- C 25	RY (11)Na 46	35.03	1.07	0.173
BD (1) O 12- C 24	RY (11)Na 46	59.65	1.07	0.226
CR (1) O 44	RY (12)Na 45	21.62	19.51	0.58
LP (1) O 44	RY (12)Na 45	16.66	0.8	0.103

LP (2) O 44	RY (12)Na 45	19.89	0.76	0.11
BD (1) H 43- O 44	RY (8)Na 45	32.29	0.93	0.155
BD (1) H 43- O 44	RY (12)Na 45	45.52	1.05	0.195
SA-$(H_2O)_2$				
CR (1) C 25	RY (7)Na 48	12.51	10.71	0.327
BD (1) O 13- C 25	RY (7)Na 48	28.25	1.24	0.167
BD (1) O 13- C 25	RY (8)Na 48	12.25	1.17	0.107
CR (1) O 10	RY (8)Na 49	8.75	19.58	0.369
CR (1) O 10	RY (9)Na 49	12.98	19.49	0.449
CR (1) O 10	RY (10)Na 49	2.53	19.48	0.198
CR (1) O 10	RY (11)Na 49	8	19.65	0.354
LP (1) O 10	LV (1)Na 49	4.46	0.61	0.046
LP (1) O 10	RY (8)Na 49	9.52	1.01	0.087
LP (1) O 10	RY (9)Na 49	14.89	0.92	0.104
BD (1) O 10- C 24	RY (8)Na 49	15.42	1.31	0.127
BD (1) O 10- C 24	RY (9)Na 49	16.86	1.22	0.128
SA-$(H_2O)_3$				
CR (1) C 24	RY (9) O 10	12.11	11.44	0.332
CR (1) C 24	RY (3) O 12	6.82	12.2	0.258
CR (1) C 24	RY (5) O 12	11.28	12.66	0.337
CR (1) C 24	RY (7) O 12	4.02	11.4	0.191
CR (1) C 24	RY (8) O 12	2.45	11.65	0.151
CR (1) C 24	RY (10) O 12	24.27	12	0.482
LP (2) O 4	BD*(1) O 5- H 37	30.66	0.84	0.143
LP (2) O 10	BD*(1) O 12- C 24	20.73	0.78	0.114
LP (2) O 10	BD*(1) C 19- C 24	18.61	0.63	0.097
LP (2) O 11	BD*(1) O 13- C 25	18.42	0.81	0.109
LP (2) O 11	BD*(1) C 22- C 25	15.63	0.62	0.088
LP (3) O 11	BD*(2) O 13- C 25	104.9	0.25	0.145
LP (2) O 12	BD*(1) O 10- C 24	19.08	0.85	0.113
LP (2) O 12	BD*(1) C 19- C 24	11.48	0.66	0.078
LP (2) O 12	RY (1) C 24	0.68	1.1	0.024
LP (2) O 12	RY (3) C 24	1.65	0.88	0.034
LP (2) O 13	BD*(1) O 11- C 25	22.13	0.78	0.117
LP (2) O 13	BD*(1) C 22- C 25	18.18	0.61	0.094
LP (2) O 5	BD*(1) H 42- O 44	11.24	0.82	0.086
LP (1) O 13	RY (12)Na 51	17.03	0.9	0.11
BD (1) O 11- C 25	RY (9)Na 51	40.56	1.06	0.185
BD (1) O 11- C 25	RY (10)Na 51	17.73	1.36	0.139
BD (1) O 11- C 25	RY (11)Na 51	12.29	1.34	0.115
BD (1) O 13- C 25	RY (9)Na 51	27.13	1.07	0.152
BD (1) O 13- C 25	RY (10)Na 51	23.86	1.38	0.162
BD (1) O 13- C 25	RY (11)Na 51	12.84	1.36	0.118
BD (1) O 13- C 25	RY (12)Na 51	22.92	1.19	0.148
CR (1) O 10	RY (12)Na 52	36.97	19.61	0.76
CR (1) O 11	RY (10)Na 52	13.53	19.72	0.461

CR (1) O 11	RY (11)Na 52	0.82	19.6	0.113
CR (1) O 11	RY (12)Na 52	39.92	19.65	0.791
CR (1) O 12	RY (12)Na 52	21.75	19.62	0.583
SA-$(H_2O)_4$				
LP (2) O 10	BD*(1) O 12- C 24	21.72	0.77	0.115
LP (2) O 10	BD*(1) C 19- C 24	19.6	0.62	0.098
LP (2) O 11	BD*(1) O 13- C 25	21.22	0.79	0.115
LP (2) O 11	BD*(1) C 22- C 25	16.93	0.6	0.09
LP (2) O 12	BD*(1) O 10- C 24	17.35	0.84	0.108
LP (2) O 12	BD*(1) C 19- C 24	15.33	0.63	0.088
LP (3) O 12	BD*(2) O 10- C 24	91.84	0.27	0.14
LP (2) O 13	BD*(1) O 11- C 25	21.57	0.83	0.12
LP (2) O 13	BD*(1) C 22- C 25	13.19	0.62	0.081
LP (3) O 13	BD*(2) O 11- C 25	108.2	0.25	0.147
BD (1) O 1- C 1	RY (7) C 14	10.42	1.29	0.103
LP (2) O 13	BD*(1) H 43- O 44	14.84	0.74	0.094
CR (1) O 10	RY (12)Na 54	27.02	19.75	0.652
CR (1) O 11	RY (5)Na 54	11.44	19.5	0.422
CR (1) O 11	RY (10)Na 54	11.09	19.62	0.416
CR (1) O 11	RY (11)Na 54	31.01	19.39	0.692
CR (1) O 12	RY (11)Na 54	12.74	19.4	0.444
CR (1) C 24	RY (12)Na 54	10.22	10.93	0.298
LP (1) O 10	RY (11)Na 54	11.61	0.82	0.087
LP (1) O 10	RY (12)Na 54	18.92	1.18	0.133
LP (1) O 11	RY (5)Na 54	12.69	0.95	0.098
LP (1) O 11	RY (10)Na 54	10.22	1.07	0.093
LP (1) O 11	RY (11)Na 54	42.28	0.83	0.167
LP (1) O 12	RY (10)Na 54	11.56	1.1	0.101
LP (1) O 12	RY (11)Na 54	26.67	0.86	0.135
BD (1) O 10- C 2	RY (12)Na 54	46.55	1.49	0.235
BD (1) O 12- C 2	RY (10)Na 54	13.72	1.35	0.122
BD (1) O 12- C 2	RY (11)Na 54	19.06	1.11	0.13
BD (1) O 12- C 2	RY (12)Na 54	21.17	1.47	0.157
LP (2) O 44	BD*(1) O 45- H 47	15.18	1	0.11
CR (1) O 44	RY (11)Na 55	12.88	19.61	0.449
LP (2) O 44	RY (11)Na 55	23.78	0.97	0.136
BD (1) H 43- O 4	RY (9)Na 55	10.63	1.12	0.097
BD (1) H 43- O 4	RY (11)Na 55	31.73	1.15	0.17
BD (1) H 43- O 4	RY (12)Na 55	9.62	1.45	0.105
LP (2) O 45	BD*(1) O 52- H 53	25.09	0.83	0.128
CR (1) O 52	RY (10)Na 55	26.11	19.45	0.636
LP (2) O 52	RY (10)Na 55	52.64	0.79	0.182
LP (2) O 52	RY (11)Na 55	8.57	0.97	0.081
LP (2) O 52	RY (12)Na 55	8.88	1.27	0.095
BD (1) H 51- O 5	RY (10)Na 55	11.58	0.98	0.095
BD (1) O 52- H 5	RY (10)Na 55	36.19	0.99	0.169
BD (1) O 52- H 5	RY (11)Na 55	10.03	1.17	0.097

BD (1) O 52- H 5	RY (12)Na 55	11.83	1.47	0.118
SA-$(H_2O)_5$				
CR (1) O 11	RY (5) C 25	14.05	20.23	0.476
CR (1) C 24	RY (4) O 12	10.81	12.55	0.329
CR (1) C 24	RY (10) O 12	16.59	11.97	0.398
CR (1) C 25	RY (10) O 11	18.98	12.12	0.428
LP (2) O 4	BD*(1) O 5- H 37	17.44	0.83	0.107
LP (2) O 9	BD*(1) O 3- C 23	20.67	0.56	0.096
LP (2) O 10	BD*(1) O 12- C 24	19.88	0.77	0.111
LP (2) O 10	BD*(1) C 19- C 24	17.28	0.64	0.094
LP (2) O 11	BD*(1) O 13- C 25	19.28	0.8	0.111
LP (2) O 11	BD*(1) C 22- C 25	15.45	0.63	0.088
LP (2) O 12	BD*(1) C 19- C 24	15.91	0.64	0.09
LP (3) O 12	BD*(2) O 10- C 24	99.22	0.26	0.143
LP (2) O 13	BD*(1) O 11- C 25	19.89	0.79	0.112
LP (2) O 13	BD*(1) C 22- C 25	17.34	0.62	0.093
CR (1) O 10	RY (9)Na 57	16.23	19.56	0.503
CR (1) O 10	RY (13)Na 57	15.97	19.69	0.501
CR (1) O 11	RY (11)Na 57	30.88	19.36	0.69
CR (1) O 12	RY (11)Na 57	15.33	19.34	0.486
LP (1) O 12	RY (11)Na 57	26.81	0.79	0.13
LP (2) O 12	RY (11)Na 57	15.57	0.49	0.078
BD (1) O 10- C 24	RY (9)Na 57	25.39	1.29	0.161
BD (1) O 10- C 24	RY (11)Na 57	26.43	1.07	0.15
BD (1) O 10- C 24	RY (13)Na 57	17.9	1.43	0.143
BD (1) O 11- C 25	RY (11)Na 57	33.27	1.08	0.17
BD (1) O 12- C 24	RY (11)Na 57	49.55	1.06	0.204
CR (1) O 11	RY (11)Na 58	28.62	19.68	0.67
CR (1) O 11	RY (13)Na 58	12.06	19.6	0.434
CR (1) O 12	RY (11)Na 58	69.88	19.67	1.046
CR (1) O 12	RY (12)Na 58	32.57	19.83	0.717
CR (1) C 25	RY (11)Na 58	17.41	10.86	0.388
LP (1) O 11	RY (11)Na 58	30.65	1.14	0.167
LP (1) O 11	RY (13)Na 58	18.97	1.05	0.126
LP (1) O 12	RY (11)Na 58	104.81	1.12	0.306
LP (1) O 12	RY (12)Na 58	38.69	1.28	0.199
LP (1) O 12	RY (13)Na 58	7.43	1.03	0.078
LP (1) O 13	RY (9)Na 58	14.39	1.02	0.108
BD (1) O 11- C 25	RY (11)Na 58	59.84	1.41	0.259
BD (1) O 11- C 25	RY (13)Na 58	15.44	1.32	0.128
BD (1) O 12- C 24	RY (11)Na 58	26.42	1.38	0.171
BD (1) O 12- C 24	RY (12)Na 58	13.42	1.54	0.128
BD (1) O 13- C 25	RY (9)Na 58	16.26	1.31	0.13
BD (1) O 13- C 25	RY (10)Na 58	6.78	1.35	0.085
BD (1) O 13- C 25	RY (11)Na 58	29.74	1.41	0.183
LP (2) O 44	BD*(1) O 45- H 47	28.89	0.84	0.139
LP (2) O 45	BD*(1) O 7- H 39	38.25	0.81	0.158

CR (1) O 54	RY (11)Na 58	39.35	19.71	0.786
CR (1) O 54	RY (12)Na 58	14.65	19.87	0.482
LP (1) O 54	RY (11)Na 58	12.93	0.98	0.101
LP (1) O 54	RY (12)Na 58	7.66	1.14	0.084
LP (2) O 54	RY (11)Na 58	62.48	0.97	0.22
LP (2) O 54	RY (12)Na 58	19.47	1.13	0.133
BD (1) O 54- H 55	RY (11)Na 58	19.71	1.24	0.139
BD (1) O 54- H 56	RY (11)Na 58	60.58	1.24	0.245
BD (1) O 54- H 56	RY (12)Na 58	13.07	1.41	0.121

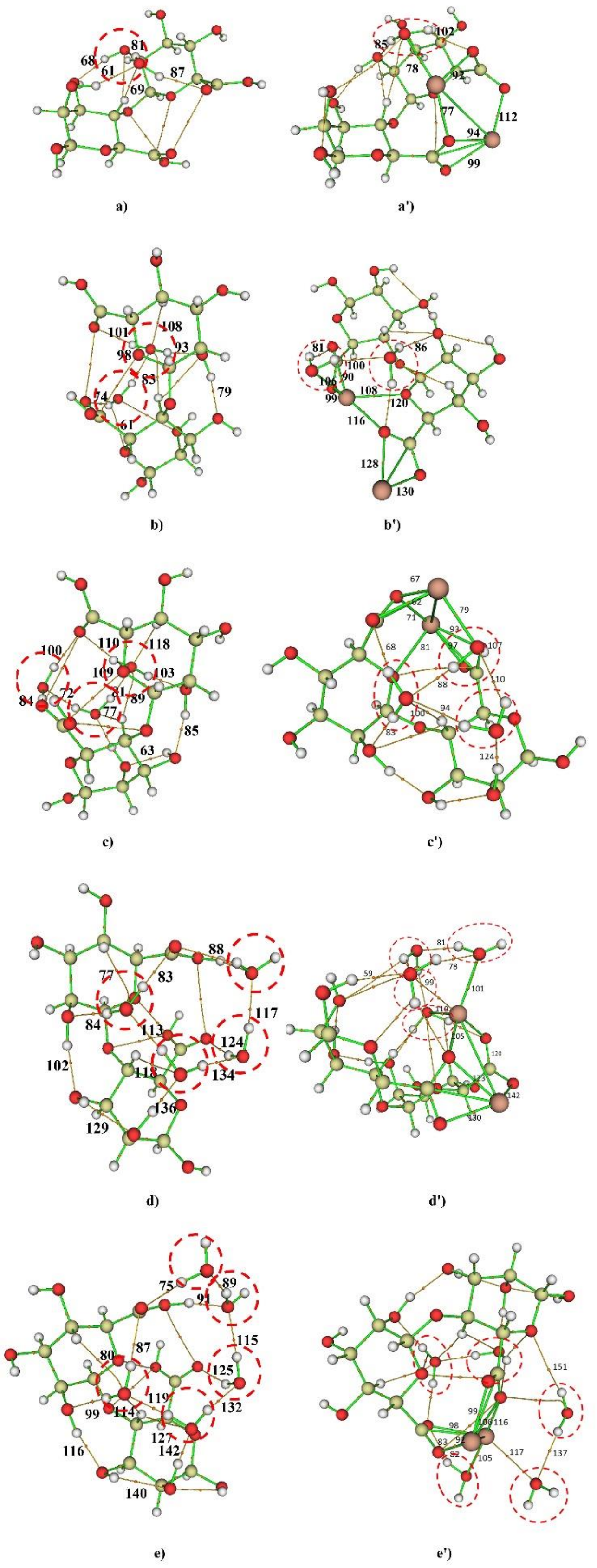

Figure S4. The molecular graphs of Alg-(H_2O)$_n$ complexes (**a-e**) and SA-(H_2O)$_n$ complexes (**a'-e'**). The BCP are represented by the unadorned orange sphere and the red circle indicates the presence of water molecule.

Table S5. The analysis of the bond critical points of the different Alg-water complexes by QTAIM

CP	BCP	$\rho_{(BCP)}$	$\nabla^2\rho_{(BCP)}$	$G_{(BCP)}$	$H_{(BCP)}$	$-V_{(BCP)}$	$\|V/G\|$	λ_1	λ_2	λ_3	$\varepsilon_{(BCP)}$	E_{HB}*
Alg-$(H_2O)_1$												
61	O4···H39	0.027	0.068	0.019	-0.002	0.020	1.085	-0.017	0.109	-0.024	0.061	-5.171
69	O46···H26	0.015	0.045	0.011	0.000	0.011	0.997	-0.015	-0.003	0.063	0.171	-2.683
81	H44···O5	0.033	0.093	0.024	-0.001	0.025	1.039	0.057	-0.033	0.069	0.073	-6.638
87	H45···O13	0.023	0.064	0.017	-0.001	0.018	1.046	0.061	0.023	-0.020	0.034	-4.399
68	H37···O4	0.041	0.114	0.030	-0.001	0.031	1.040	-0.055	0.185	-0.016	0.041	-8.299
Alg-$(H_2O)_2$												
74	H48···O12	0.020	0.055	0.015	-0.001	0.016	1.058	-0.006	-0.007	0.068	0.027	-3.771
61	H48···O3	0.011	0.042	0.009	0.001	0.008	0.894	-0.004	0.010	0.037	0.467	-1.663
77	H49···O46	0.034	0.095	0.025	-0.001	0.026	1.051	0.011	0.131	-0.047	0.233	-6.906
108	O46···H33	0.008	0.028	0.006	0.001	0.005	0.836	-0.005	0.030	0.002	0.175	-1.026
83	O46···H26	0.007	0.029	0.006	0.001	0.005	0.776	-0.005	0.009	0.024	1.129	-0.929
93	H44···O5	0.034	0.095	0.025	-0.001	0.026	1.033	0.090	-0.044	0.050	0.069	-6.792
98	H45···O2	0.007	0.032	0.007	0.001	0.005	0.786	-0.002	-0.004	0.038	3.406	-0.734
101	H45···O13	0.023	0.063	0.017	-0.001	0.018	1.052	0.062	0.005	-0.004	0.029	-4.455
79	H37···O4	0.040	0.126	0.031	0.000	0.031	0.990	-0.052	0.196	-0.018	0.070	-8.079
Alg-$(H_2O)_3$												
110	H45···O13	0.022	0.061	0.016	-0.001	0.017	1.051	0.074	-0.006	-0.006	0.031	-4.232
109	H45···O2	0.007	0.032	0.006	0.001	0.005	0.783	-0.002	-0.004	0.037	2.152	-0.722
103	H44···O5	0.036	0.100	0.026	-0.001	0.027	1.033	0.087	-0.045	0.059	0.055	-7.185
118	O46···H33	0.011	0.034	0.008	0.001	0.007	0.905	-0.009	0.039	0.004	0.102	-1.603
89	O46···H26	0.007	0.025	0.005	0.001	0.004	0.786	-0.005	0.008	0.022	0.175	-0.769
72	H48···O50	0.041	0.112	0.030	-0.002	0.031	1.051	0.178	-0.033	-0.033	0.107	-8.366
81	H49···O46	0.042	0.118	0.031	-0.001	0.032	1.043	-0.054	0.227	-0.054	0.045	-8.634
77	O47···H26	0.004	0.015	0.003	0.001	0.002	0.696	-0.002	0.002	0.015	1.325	-0.124
100	H52···O13	0.014	0.049	0.012	0.000	0.011	0.967	-0.011	0.066	-0.006	0.049	-2.471
84	H51···O12	0.033	0.100	0.025	0.000	0.025	0.997	-0.046	-0.039	0.185	0.011	-6.556
85	H37···O4	0.038	0.105	0.027	-0.001	0.028	1.035	-0.050	0.206	-0.051	0.044	-7.731
63	H36···O7	0.029	0.090	0.024	-0.001	0.025	1.053	0.039	0.057	-0.006	0.204	-5.769

Table S5. (*Continued*)

CP	BCP	$\rho_{(BCP)}$	$\nabla^2\rho_{(BCP)}$	$G_{(BCP)}$	$H_{(BCP)}$	$-V_{(BCP)}$	$\|V/G\|$	λ_1	λ_2	λ_3	$\varepsilon_{(BCP)}$	E_{HB}*
Alg-$(H_2O)_4$												
113	O46···H49	0.041	0.116	0.030	-0.001	0.313	10.375	-0.018	0.195	-0.061	0.045	-8.443
83	H45···O13	0.024	0.069	0.018	-0.001	0.018	1.032	0.062	0.008	-0.001	0.043	-4.658
84	H44···O5	0.033	0.091	0.024	-0.001	0.025	1.040	0.092	-0.034	0.033	0.059	-6.529
91	O54···H43	0.047	0.131	0.034	-0.001	0.036	1.042	0.012	0.014	0.105	0.024	-9.713
117	O54···H52	0.031	0.089	0.023	-0.001	0.024	1.036	-0.004	0.136	-0.043	0.033	-6.280
124	H51···O12	0.032	0.095	0.024	0.000	0.024	1.002	-0.022	-0.010	0.127	0.008	-6.301
117	H52···O54	0.051	0.141	0.038	-0.003	0.040	1.066	-0.029	0.178	-0.008	0.056	-10.669
134	O50···H48	0.042	0.115	0.030	-0.001	0.032	1.047	0.159	-0.058	0.014	0.026	-8.583
102	H37···O4	0.036	0.100	0.026	-0.001	0.027	1.037	-0.043	0.197	-0.055	0.049	-7.344
129	H36···O7	0.026	0.082	0.021	-0.001	0.022	1.037	0.039	0.060	-0.017	0.292	-4.976
Alg-$(H_2O)_5$												
119	O46···H49	0.042	0.120	0.031	-0.001	0.032	1.036	0.015	0.166	-0.062	0.046	-8.656
87	H45···O13	0.018	0.050	0.013	-0.001	0.013	1.041	0.013	0.024	0.012	0.038	-3.196
99	H44···O5	0.032	0.088	0.023	-0.001	0.024	1.044	0.131	-0.044	0.002	0.058	-6.369
142	O47···H39	0.056	0.151	0.042	-0.004	0.046	1.094	0.130	0.106	-0.084	0.042	-11.718
132	H48···O50	0.042	0.117	0.031	-0.001	0.032	1.044	0.133	-0.065	0.049	0.028	-8.697
125	H51···O12	0.031	0.093	0.023	0.000	0.024	1.012	0.004	-0.025	0.114	0.015	-6.139
115	H52···O54	0.033	0.093	0.024	-0.001	0.025	1.038	-0.022	0.144	-0.029	0.037	-6.650
91	O54···H43	0.067	0.157	0.049	-0.010	0.059	1.202	0.227	-0.033	-0.038	0.038	-14.203
89	H55···O56	0.047	0.131	0.035	-0.002	0.037	1.059	-0.077	0.127	0.080	0.042	-9.830
75	H58···O13	0.032	0.094	0.024	0.000	0.024	1.003	0.155	-0.042	-0.019	0.019	-6.400
116	H37···O4	0.034	0.092	0.024	-0.001	0.025	1.042	-0.048	0.189	-0.050	0.048	-6.824
140	H36···O7	0.022	0.076	0.019	0.000	0.019	0.995	0.059	0.032	-0.015	0.538	-4.109

CP: critical point; BCP: Bond critical point); ρ: Electron density (a.u.); $\nabla^2\varrho$: Laplacian of electron density (a.u); G: Lagrangian kinetic energy (a.u); H: Hamiltonian kinetic energy or electronic energy density (a.u); V: Potential energy density (a.u); ε: Ellipticity of electron density (a.u); λ_1, λ_2, λ_3 : Components of Laplacian in x/y/z (a.u); E_{HB}: Hydrogen bond energy (kcal/mol)

Table S6. The analysis of the bond critical points of the different SA-water complexes by QTAIM

CP	BCP	$\rho_{(BCP)}$	$\nabla^2\rho_{(BCP)}$	$G_{(BCP)}$	$H_{(BCP)}$	$-V_{(BCP)}$	$\lvert V/G \rvert$	λ_1	λ_2	λ_3	$\varepsilon_{(BCP)}$
SA-$(H_2O)_1$											
78	O44… Na45	0.024	0.145	0.031	0.006	0.025	0.815	0.159	-0.013	-0.002	0.049
92	O13…Na45	0.021	0.130	0.027	0.005	0.022	0.807	-0.024	0.179	-0.025	0.087
77	O12…Na45	0.028	0.190	0.039	0.008	0.031	0.784	0.050	-0.006	0.146	0.045
112	O11…Na46	0.028	0.192	0.039	0.009	0.031	0.783	-0.002	0.152	0.042	0.077
94	O12⋯NA46	0.022	0.131	0.028	0.005	0.022	0.814	0.005	0.100	0.025	0.044
99	O10…Na46	0.023	0.143	0.030	0.006	0.025	0.813	0.008	0.107	0.029	0.014
85	O13…H43	0.019	0.051	0.014	-0.001	0.015	1.067	0.009	-0.022	0.064	0.072
102	O5…H42	0.027	0.081	0.021	-0.001	0.022	1.041	0.083	0.008	-0.009	0.127
SA-$(H_2O)_2$											
130	Na48…O11	0.027	0.177	0.037	0.007	0.029	0.800	0.133	-0.013	0.057	0.014
128	Na48…O13	0.027	0.179	0.037	0.008	0.030	0.798	0.001	0.210	-0.032	0.007
120	H43…O13	0.028	0.075	0.020	-0.001	-0.001	0.054	-0.019	0.012	0.082	0.079
116	Na49…O13	0.022	0.133	0.028	0.005	0.022	0.806	0.007	0.150	-0.025	0.064
108	O2…Na49	0.010	0.053	0.011	0.002	0.010	0.837	0.065	-0.009	-0.003	0.110
100	H47…o44	0.024	0.064	0.017	-0.001	0.019	1.083	0.116	-0.024	-0.028	0.116
90	O12…Na49	0.021	0.129	0.027	0.001	0.022	0.813	-0.023	0.157	-0.005	0.133
99	O10…Na49	0.022	0.139	0.029	0.006	0.024	0.808	-0.021	0.095	0.065	0.074
81	O12…H46	0.027	0.078	0.029	0.020	0.001	0.029	0.014	0.037	0.028	0.114
106	Na49…O45	0.020	0.115	0.025	0.004	0.021	0.833	0.018	-0.023	0.120	0.063
86	O5…H42	0.009	0.030	0.007	$4x10^{-4}$	0.007	0.938	0.0310	$-2.3x10^{-5}$	-0.001	0.075

Table S6. (*Continued*)

CP	BCP	$\rho_{(BCP)}$	$\nabla^2\rho_{(BCP)}$	$G_{(BCP)}$	$H_{(BCP)}$	$-V_{(BCP)}$	$\lvert V/G \rvert$	λ_1	λ_2	λ_3	$\varepsilon_{(BCP)}$
SA-$(H_2O)_3$											
110	H46…O48	0.021	0.060	0.016	-0.001	0.016	1.039	0.025	0.040	-0.005	0.136
94	O44…H47	0.020	0.057	0.015	-0.001	0.016	1.046	-0.022	0.090	-0.010	0.145
124	H39…O45	0.046	0.132	0.034	-0.001	0.036	1.038	0.056	0.000	0.076	0.041
79	048…Na51	0.026	0.165	0.035	0.007	0.028	0.812	0.020	0.179	-0.033	0.053
81	O2…Na52	0.010	0.054	0.012	0.002	0.010	0.843	0.062	-0.010	0.001	0.094
71	O11…Na52	0.026	0.168	0.035	0.007	0.028	0.794	-0.034	0.078	0.124	0.060
67	O11…Na51	0.020	0.122	0.026	0.005	0.021	0.819	-0.009	0.031	0.100	0.056
62	O13…Na51	0.022	0.139	0.029	0.006	0.024	0.810	0.120	0.045	-0.026	0.024
88	H50…o44	0.034	0.092	0.024	-0.001	0.025	1.046	0.139	-0.009	-0.037	0.037
107	H49…O12	0.049	0.149	0.038	-0.001	0.040	1.032	-0.078	-0.081	0.307	0.035
93	Na52…O12	0.021	0.132	0.028	0.005	0.022	0.808	-0.023	0.128	0.027	0.123
97	Na52…O10	0.026	0.171	0.036	0.007	0.028	0.798	-0.010	0.160	0.022	0.033
100	O44…H26	0.006	0.023	0.005	0.001	0.004	0.783	-0.004	0.005	0.022	0.673
68	H43…O13	0.028	0.081	0.021	-0.001	0.022	1.030	0.035	0.063	-0.016	0.066
83	O5…H42	0.029	0.079	0.021	-0.001	0.022	1.047	0.068	-0.038	0.050	0.061
SA-$(H_2O)_4$											
120	Na55…O13	0.294	-1.100	0.038	-0.313	0.351	9.210	-0.651	-0.572	0.124	0.035
101	Na55…O52	0.010	0.028	0.007	0.000	0.007	1.022	-0.005	0.038	-0.005	0.213
105	NA55…O12	0.017	0.096	0.021	0.003	0.017	0.832	0.019	0.045	0.032	0.063
110	Na55…O44	0.005	0.021	0.004	0.001	0.002	0.642	0.003	0.008	0.009	-1.657
130	Na54…O10	0.324	-1.868	0.071	-0.538	0.608	8.624	-1.261	1.047	-1.655	0.019
123	O12…Na54	0.045	0.126	0.033	-0.002	0.035	1.047	-0.029	0.225	-0.070	0.034
142	Na54…O11	0.018	0.118	0.024	0.006	0.018	0.747	0.063	0.051	0.004	-1.213
81	O45…H53	0.262	-0.582	0.227	-0.372	0.599	2.641	0.228	-0.532	-0.278	0.183
99	H47…O44	0.029	0.195	0.041	0.008	0.032	0.799	0.106	0.111	-0.021	0.040
59	H41…O48	0.358	-2.087	0.073	-0.594	0.667	9.196	0.078	-0.982	-1.183	0.025
78	H50…O52	0.007	0.032	0.006	0.002	0.005	0.724	0.011	0.020	0.002	-2.030

Table S6. (*Continued*)

CP	BCP	$\rho_{(BCP)}$	$\nabla^2\rho_{(BCP)}$	$G_{(BCP)}$	$H_{(BCP)}$	$-V_{(BCP)}$	$\|V/G\|$	λ_1	λ_2	λ_3	$\varepsilon_{(BCP)}$
SA-$(H_2O)_5$											
117	(Na58…O52)	0.007	0.055	0.008	0.063	0.006	0.818	-0.006	-0.007	0.068	0.082
105	(Na58…O54)	0.017	0.096	0.021	0.116	0.017	0.832	0.019	0.045	0.032	0.063
98	(Na58…O13)	0.017	0.096	0.021	0.117	0.017	0.830	0.037	0.065	-0.006	0.180
83	(O13…H56)	0.029	0.083	0.022	0.105	0.023	1.054	0.128	-0.037	-0.008	0.100
92	(Na58…O11)	0.021	0.131	0.027	0.158	0.022	0.809	-0.017	0.100	0.048	0.030
116	(Na58…O12)	0.024	0.168	0.034	0.201	0.026	0.758	0.032	0.043	0.092	0.048
106	(O12…Na57)	0.016	0.089	0.019	0.108	0.016	0.839	-0.003	0.047	0.045	0.377
99	(O10…Na57)	0.029	0.195	0.041	0.236	0.032	0.799	0.106	0.111	-0.021	0.040
82	(Na57…O11)	0.027	0.185	0.038	0.223	0.030	0.779	-0.033	0.073	0.145	0.052
137	(H50…O52)	0.013	0.037	0.010	0.047	0.010	1.022	0.009	0.036	-0.008	0.077
151	(H49…O3)	0.003	0.015	0.003	0.018	0.002	0.674	0.018	-0.003	-0.0003	0.156

CP: critical point; BCP: Bond critical point); ρ: Electron density (a.u.); $\nabla^2\varrho$: Laplacian of electron density (a.u); G: Lagrangian kinetic energy (a.u); H: Hamiltonian kinetic energy or electronic energy density (a.u); V: Potential energy density (a.u); ε: Ellipticity of electron density (a.u); λ_1, λ_2, λ_3 : Components of Laplacian in x/y/z (a.u); E_{HB}: Hydrogen bond energy (kcal/mol)

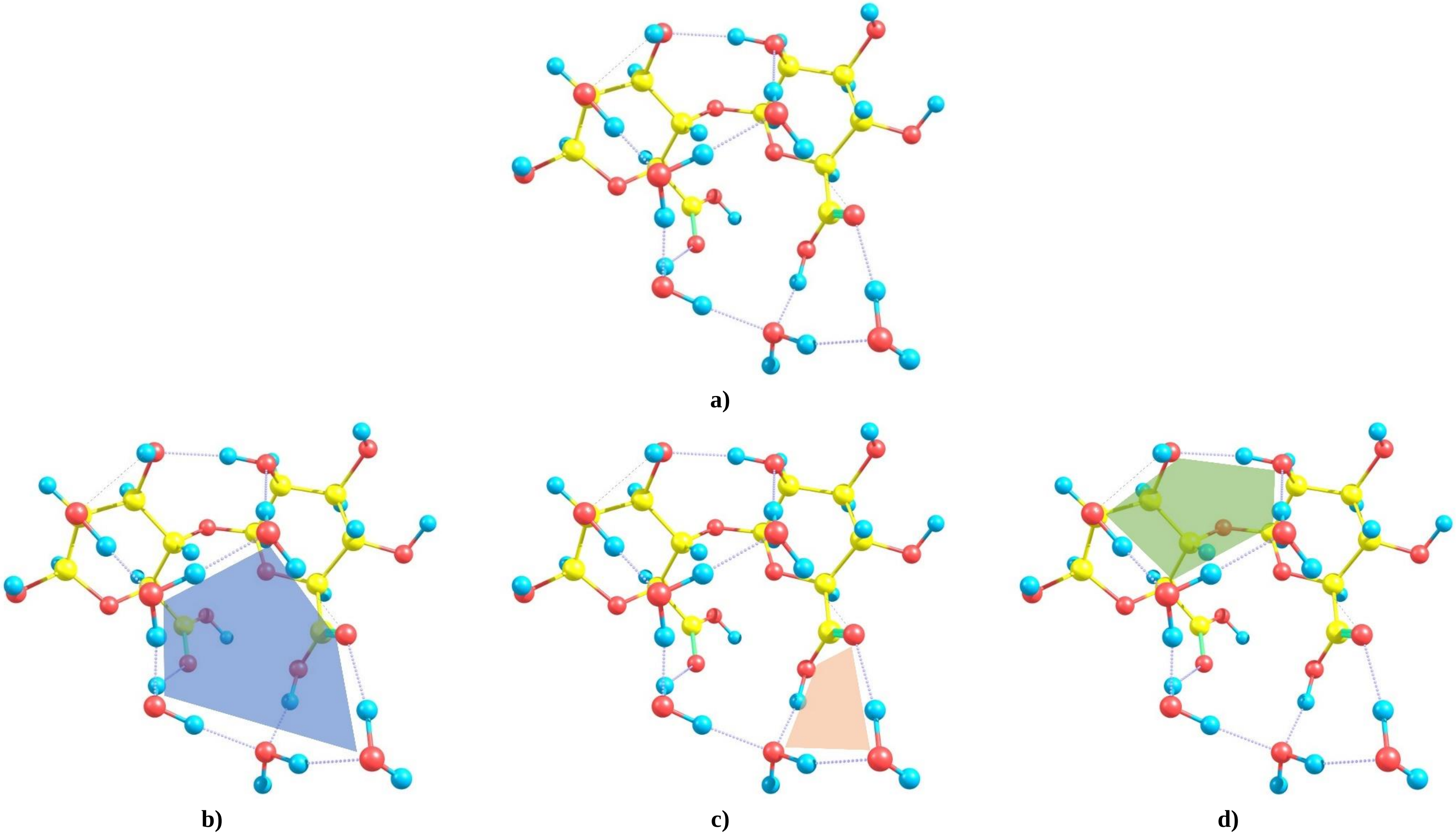

Figure S5. Optimized structure of Alg-$(H_2O)_5$ **(a)** and cyclic on Alg-(H_2O) structure **(b-d)**
